# Affinity-Based Detection of Biomolecules Using Photo-Electrochemical Readout

**DOI:** 10.3389/fchem.2019.00617

**Published:** 2019-09-11

**Authors:** Amanda Victorious, Sudip Saha, Richa Pandey, Tohid F. Didar, Leyla Soleymani

**Affiliations:** ^1^School of Biomedical Engineering, McMaster University, Hamilton, ON, Canada; ^2^Department of Engineering Physics, McMaster University, Hamilton, ON, Canada; ^3^Department of Mechanical Engineering, McMaster University, Hamilton, ON, Canada

**Keywords:** biosensing, photoelectrochemical (PEC), affinity-based bio, photoactive materials, plasmonic biosensing

## Abstract

Detection and quantification of biologically-relevant analytes using handheld platforms are important for point-of-care diagnostics, real-time health monitoring, and treatment monitoring. Among the various signal transduction methods used in portable biosensors, photoelectrochemcial (PEC) readout has emerged as a promising approach due to its low limit-of-detection and high sensitivity. For this readout method to be applicable to analyzing native samples, performance requirements beyond sensitivity such as specificity, stability, and ease of operation are critical. These performance requirements are governed by the properties of the photoactive materials and signal transduction mechanisms that are used in PEC biosensing. In this review, we categorize PEC biosensors into five areas based on their signal transduction strategy: (a) introduction of photoactive species, (b) generation of electron/hole donors, (c) use of steric hinderance, (d) in situ induction of light, and (e) resonance energy transfer. We discuss the combination of strengths and weaknesses that these signal transduction systems and their material building blocks offer by reviewing the recent progress in this area. Developing the appropriate PEC biosensor starts with defining the application case followed by choosing the materials and signal transduction strategies that meet the application-based specifications.

## Introduction

Biosensors are devices that are used for analyzing biologically-relevant species using specific biorecognition elements and transducers (Soleymani and Li, 2017). Based on the nature of the biorecognition event, biosensors are classified into biocatalytic and affinity-based devices (Zhao et al., 2015b). In biocatalytic biosensors, immobilized enzymes are used to recognize their specific substrate molecule, whereas affinity-based biosensors incorporate a synthetic or biological capture agent such as aptamers (Zhao et al., 2016), DNAzyme (Zhao et al., 2017a), single stranded DNA (Zhao et al., 2014), or antibodies (Zhao et al., 2018) to specifically capture the biologically-relevant target. The interaction between the analyte and the capture agent is translated into a readable signal by a transducer. To date, transduction methods relying on acoustic (Zhang et al., 2018d), optical (Špačková et al., 2016), gravimetric (DeMiguel-Ramos et al., 2017), electrochemical (Alizadeh and Salimi, 2018), electronic (Kirste et al., 2015), and photoelectrochemical mechanisms (Zhao et al., 2015b) have been reported for use in biosensing systems. Researchers often choose a transduction method that offers the right level of sensitivity, specificity, speed, and multiplexing for the desired application, and meets requirements with respect to instrumentation cost, size, and ease-of-use.

Due to the growing demand for rapid clinical diagnosis and health monitoring using handheld systems, there has been an increasing push for the development of new bioanalytical techniques that combine high sensitivity, specificity, and speed with portable and inexpensive readout instrumentation. Photoelectrochemistry is an emerging signal transduction method that has the potential to meet the stringent requirements of the field of biosensing. In photoelectrochemical (PEC) bioanalysis, biological interactions between the analyte and the biorecognition element result in a change in the generated PEC current or voltage. In these systems, the photo-electrode or PEC label used in the biosensor is activated upon optical excitation. This optical excitation or biasing reduces the reliance of PEC systems on electrical biasing, which allows them to be operated under low or no applied electric potential. It has been shown that a lower limit-of-detection can be achieved using PEC signal readout compared to a similar assay that is coupled to electrochemical readout (Yildiz et al., 2008; Golub et al., 2009). Although PEC biosensors rely on *both* optical and electrochemical mechanisms, they can be excited using low powered broad-spectrum light sources and read using inexpensive electrical circuits. As a result, it is possible to miniaturize PEC systems into inexpensive and integrated platforms that are similar in operation to handheld electrochemical readers (Golub et al., 2009). Additionally, PEC biosensors can be easily multiplexed by incorporating multiple individually accessible electrodes on the same platform.

**Figure 1:**
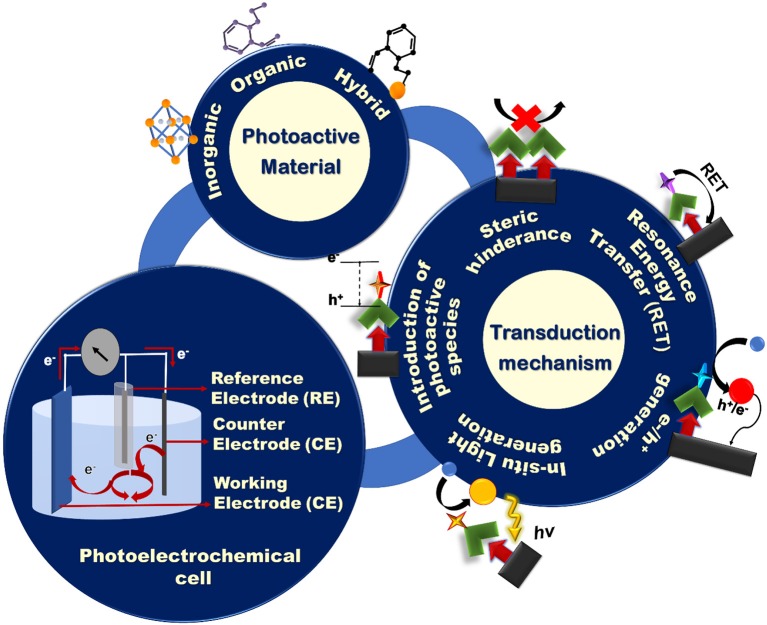
Schematic representation of the building blocks of a PEC biosensing system: the PEC cell, photoactive materials, and various signal transduction architectures.

*Affinity-based* PEC biosensors combine the high specificity of biorecognition agents such as ssDNA, antibodies, and aptamers, with the sensitivity of PEC biosensors, and are the focus of this review article. There are previously-published review articles that are focused on a specific type of biorecognition-target interaction such as DNA sensing (Zhao et al., 2014), immunoassays (Zhao et al., 2018), enzymatic sensing (Zhao et al., 2017a), and aptasensing (Deng et al., 2016; Zhao et al., 2016). However, our focus is on the elements that are important for building a PEC biosensor, regardless of the target analyte. Toward this goal we will discuss the construction of a photoelectrochemical cell, photoactive materials used in creating these devices, and the signal transduction mechanisms that are employed in PEC signal generation (Figure 1).

## Construction of a Photoelectrochemical Cell

Generally, a PEC cell consists of an optical excitation source, an electrochemical cell, and an electrochemical reader. The electrochemical cell consists of four main components (Figure 1): (i) a working electrode (WE) that is often constructed by immobilizing photoactive materials on a conductive substrate, (ii) a counter electrode (CE), (iii) a reference electrode, and (iv) an electrolyte to generate PEC signals using redox reactions. Upon illumination, the redox reactions driven by the electrochemically active species in the electrolyte generate an electric signal between the WE and the CE that is recorded by the electrochemical reader. To create an application-specific PEC biosensor, much attention has to be paid to the design of: (i) the sensing electrodes using photoactive species having the appropriate electronic and optical properties and/or conductive collectors; (ii) the transduction mechanism based on the target analyte and device application; and (iii) the electrolyte that contains the redox species that participate in the generation of the photoelectrochemical signal. The majority of the affinity-based PEC biosensing strategies reported to date rely on measuring photocurrents for signal readout (Zhao et al., 2018). To design a PEC bioassay, suitable for a specific application, it is important to have a comprehensive knowledge of these components and the strategies that are used in incorporating them in a synergistic fashion.

## Photoactive Species for PEC Biosensors

Photoactive species are materials that respond to optical excitation by generating excited electronic states and converting optical energy to chemical and electrical energy (Bard et al., 1980). These species enable a PEC cell to generate or modify an electrochemical signal in response to light or electromagnetic radiation. In PEC biosensors, photoactive species are used as the building blocks of photoactive electrodes and/or as labels or reporters that associate with the biorecognition element (Fan et al., 2015), target analyte (Han et al., 2017a), or solution-borne surfaces such as magnetic beads and metallic nanoparticles (NPs) (Tu et al., 2018). Due to its instrumental role in signal transduction, choosing the right photoactive material is critical to the development of PEC biosensors.

The photoactive materials used in PEC biosensing are chosen based on their electronic and optical parameters (incident photon-to-current conversion efficiency (IPCE), carrier mobility, response time, energy levels, and absorption spectrum), size/structure, stability against photobleaching, and ability to functionalize and integrate into devices. One of the most important parameters for evaluating photoactive materials used in PEC devices is IPCE. IPCE measures the photocurrent collected per incident photon flux as a function of illumination wavelength, which allows researchers to compare the efficiency of the photoactive species at different regions of the electromagnetic spectrum (Chen et al., 2013). IPCE *collectively* evaluates the optical and electronic properties of materials such as their ability to absorb electromagnetic radiation and transport and collect charged carriers through the PEC cell. The electronic and optical properties of photoactive materials need to be selected such that the materials can supply charge carriers having sufficient energy (indicated by the band structure of the material) to drive the desired electrochemical reaction. It is also important for these electrochemical reactions to occur at high rates (measured using IPCE). The wavelength dependence of IPCE is important in understanding the type of optical excitation source that is required for designing a PEC biosensor (Kolesova et al., 2018). Fine-tuning the size and shape of photoactive species in the nanoscale is also important for enhancing the PEC performance of the biosensor as structural tunability on the nanoscale changes the band structure of the materials, and can be used to enhance the surface-to-volume ratio of electrodes created from photo-active materials (Fu and Zhang, 2018). Resilience to photobleaching is important because photoactive materials that degrade due to multiple cycles of photoinduction do not allow the target-induced changes in the photocurrent to be reliably measured in a PEC biosensor (Kolesova et al., 2018). Furthermore, for a photoactive material with the desired electronic, optical, and stability parameters to be used in a biosensing device, it is critical for it to have a chemical structure that can be easily functionalized with the typical termination chemistries of biorecognition elements (amine, thiol, carboxyl, aldehyde, to name a few). Finally, it is critical for these photoactive materials to have the sufficient level of mechanical robustness and adhesion to be integrated into miniaturized chips or strips used in biosensing platforms.

The three major classes of photoactive materials commonly used in PEC biosensors include (i) inorganic semiconductors, (ii) organic semiconductors, and (iii) hybrid semiconductors (Devadoss et al., 2015; Zhu et al., 2016), which will be discussed in detail in this section.

### Inorganic Semiconductors

**Figure 2:**
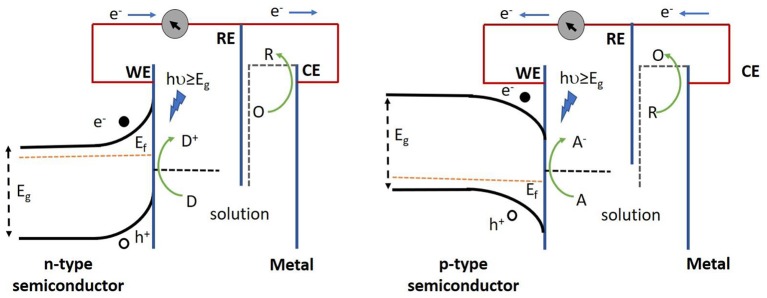
Operation of n-type semiconductor (left) and p-type semiconductor (right) in a PEC cell. Electron donors (D) in the redox couple are oxidized by the photoactive species, thereby resulting in the loss of an electron which is then relayed to the collector (underlying conductive substrate), thus yielding an anodic current. Alternatively, electron acceptors (A) in the redox couple gain an electron from the photoactive species following their reduction upon light illumination, subsequently giving rise to a cathodic current. E_g_, E_f_, WE, RE, CE represent band gap energy, fermi energy, working electrode, reference electrode and counter electrode respectively.

Semiconductors from non-carbonous materials are known as inorganic semiconductors. Generally, in inorganic semiconductor transducers, electrons are excited from the valence band (VB) to the conduction band (CB) upon absorption of photons with energies higher than that of their band gaps. This results in the generation of electron-hole pairs that can engage in redox reactions at the surface of the working electrode. The direction of the photocurrent (anodic or cathodic) depends on the applied electric field and the position of the semiconductor Fermi level with respect to the electrochemical potential of the electrolyte (Figure 2). In general, the mobile charge carriers (electrons for n-type and holes for p-type semiconductors) in the semiconductor traverse the bulk of the electrode while minority carriers take part in the redox reactions at its surface (Gratzel, 2001). Therefore, usually n-type semiconductors are used to produce anodic photocurrents, whereas p-type semiconductors are chosen for cathodic photocurrent generation (Zhao et al., 2014).

Inorganic semiconductors such as titanium dioxide (TiO_2_) (Tian et al., 2018), cadmium telluride (CdTe) (Lin et al., 2016; Hao et al., 2017b; Li et al., 2018a), cadmium sulfide (CdS) (Gong et al., 2015; Ge et al., 2016a; Lv et al., 2017), Molybdenum disulphide (MoS_2_) (Zang et al., 2016; Wang et al., 2019), cadmium selenide (CdSe) (Matylitsky et al., 2009; Wang et al., 2014c), and zinc oxide (ZnO) (Han et al., 2017b) are used extensively in affinity based PEC biosensors (Hong et al., 2015; Lin et al., 2016; Hao et al., 2017b; Li et al., 2018a). Inorganic semiconductors offer a few advantages over their organic counterparts as discussed in the following section. These materials typically exhibit longer stability under mechanical, electrical, and environmental stress (Yu et al., 2017b). High-performance inorganic semiconductors can be precisely fabricated into various structures at the nanoscale, matching the size of subcellular and molecular components and allowing better probing of biological targets (Jiang and Tian, 2018). Inorganic semiconductors usually require lower bias voltages (due to their higher charge mobility and charge-carrier separation efficiency) (Nelson, 2002), exhibit faster response time in the generation of charge carriers upon excitation, and allow for easier device passivation for use in physiological fluids as compared to organic SCs (Jiang and Tian, 2018).

Photochemical stability and high catalytic efficiency make TiO_2_ a promising material for affinity-based PEC biosensing (Tomkiewicz and Woodall, 1977; Wang et al., 2014b). While promising, pristine TiO_2_ suffers from a variety of problems such as poor response in the visible range owing to its wide band gap (Liu et al., 2010) and relatively fast recombination of photogenerated charge carriers (Chen and Mao, 2007). In order to overcome these limitations, scientists are turning to hybrid TiO_2_ architectures that incorporate other materials such as metal cations and anions (Daghrir et al., 2013), semiconductors of M_x_S_y_ (Du et al., 2017) and M_x_O_y_ (Liu et al., 2017a) configuration, and carbon based materials (Komathi et al., 2016). CdTe is also widely used in PEC biosensing owing to its large bulk absorption coefficient (>10^4^ cm^−1^ in the red, ~10^5^ cm^−1^ in the blue) (Mitchell et al., 1977) and near-infrared band gap (*E*_*g*_ ≈ 1.5 eV) (Sarkar et al., 2012) making it suitable for operation in the visible region of the solar spectrum. One of the issues with CdTe is the low abundance of tellurium, which makes it economically inviable for biosensing (Wang et al., 2010). Due to the availability of precursors and ease of crystallization (Zhou et al., 2015), there is a move toward other chalcogenides of CdE (E = S, Se). These alternatives are mostly used as sensitizers due to their narrow band gaps (CdS = 2.4 eV, CdSe = 1.7 eV) (Zhou et al., 2015). In addition, they offer higher conduction bands edges compared to most metal oxides (ZnO, TiO_2_), making them useful for reactions where electrons need to be transferred from the conduction band of these materials (Zhou et al., 2015). However, their inability to integrate into device fabrication due to their poor adhesion onto the substrate and the inherent toxicity of Cd limit the possibility of using Cd-based materials in commercial biosensing platforms (Yue et al., 2013). ZnO is another wide band gap (direct band gap of 3.37 eV in the near UV spectral region) semiconductor used in PEC biosensors, which offers biocompatibility, excellent photoactivity (large exciton binding energy at room temperature (Özgür et al., 2005), high charge carrier mobility, and thermal and chemical stability (Tu et al., 2011). ZnO can be structurally tuned and has been used in flower (Han et al., 2017a), rod (Kang et al., 2015), wire (Zhao et al., 2016), and pencil (Qiao et al., 2016) architectures for biosensing applications. Nevertheless, the photocatalytic performance of ZnO diminishes in aqueous solutions due to the fast recombination of photogenerated charge carriers (Liu et al., 2014). Apart from these materials, emerging inorganic semiconductors are being investigated for use in PEC biosensing. Specifically, MoS_2_ is under investigation for use in PEC cells due to its ability to generate an internal electric field at the contact surface for photoinduced charge separation, which increases the carrier lifetime (Jiang et al., 2019). Additionally, Bi-X (X = S,V,O) materials are being investigated due to their tunable bandgap and photostability in acidic solutions (Jiang et al., 2019).

Inorganic semiconductors are widely used in developing PEC biosensors; however, a remaining challenge, as with other types of materials used in biosensors is related to non-specific adsorption causing interference to the signal by producing high noise levels or low reactivity (Rim et al., 2017). Hydrophilic coating strategies have been employed for metal oxides and sulfides but most of these strategies have downsides under severe biological conditions or where long-term stability is needed. Furthermore, conditions such as high temperature, high salinity, and non-neutral pH, intensify these effects (Cheng et al., 2008; Kadian et al., 2018). To overcome these challenges, researchers are focusing on integrating inorganic semiconductors into hybrid antifouling networks, which has been previously reviewed (Zhao et al., 2016).

### Organic Semiconductors

Organic materials such as graphitic carbon nitride (g-C_3_N_4_), porphyrin, azo dyes, chlorophyll, bacteriorhodopsin, and polymers such as semiconducting polymer dots (Pdot), phthalocyanine, poly(thiophene), phenylenevinylene (PPV), and their derivatives have been used for constructing photoactive electrodes that can be applied to PEC biosensors (Ikeda et al., 2009; Da et al., 2018; Shi et al., 2018b). Some of the main advantages offered by organic semiconductors lies in their improved mechanical compliance (Xu et al., 2017), intrinsic stretchability (Xu et al., 2017), and their amenability to low-temperature all-solution-based processing (Zhao et al., 2017b; Jiang and Tian, 2018). This allows inexpensive fabrication of large-area films on flat, irregular, and flexible substrates, which provides opportunities for the development of flexible and printed electronic based biosensors used in wearable technology (Malliaras, 2001; Zhao et al., 2017b).

Graphitic carbon nitride (g-C_3_N_4_) is a metal-free two dimensional polymeric semiconductor, which is attractive for PEC biosensing due to its high physicochemical stability and inexpensive and earth abundant nature (Wang et al., 2017). It has a smaller band gap (~2.7 eV) (Su et al., 2010) compared to commercial TiO_2_ NPs (~3.0-3.2 eV) and is able to absorb light in the visible portion of the solar spectrum up to 460 nm (Ong et al., 2016). G-C_3_N_4_ has a desirable electronic band structure due to the presence of π-conjugated sp^2^ hybridized carbon and nitrogen. It is also electron-rich and has basic surface functionalities due to the presence of Lewis and Brönstead basic functions (Zhu et al., 2014). This has enabled g-C_3_N_4_ to be applied to the degradation of organic pollutants, hydrogen evolution reaction, biosensing, and energy conversion (Wu et al., 2015; Liu and Dai, 2016; Li et al., 2017a; Panneri et al., 2017; Tong et al., 2017; Shi et al., 2018a). Da et al. (2018) constructed a novel “signal-off” PEC aptasensor using an aptamer bridged DNA network in conjunction with g-C_3_N_4_ to detect vascular endothelial growth factor (VEGF165). This photo-electrode exhibited a stable photocurrent response with no severe decay under periodic off-on-off light excitation for nine cycles over a timeframe of 350 s. While g-C_3_N_4_ is a promising material, its low quantum yield in its pristine form (0.1% at 420–460 nm) (Maeda et al., 2009) remains an obstacle to its incorporation as a high-performance photoactive material for biosensing (Zheng et al., 2012; Zhang et al., 2013). In order to enhance the efficiency of this material, the use of g-C_3_N_4_ in conjunction with materials like TiO_2_ and CdS has been reported for biosensing applications (Liu et al., 2015; Wang et al., 2016a; Fan et al., 2018). The formation of heterojunction with these materials helps to accelerate the charge transport and reduce the recombination rate by separating the charge carriers generated in g-C_3_N_4_ (Ong et al., 2016).

Porphyrins, a group of macrocyclic organic compounds composed of four pyrrole rings joined via methine (= CH-) bonds are being investigated for use as photoactive materials or sensitizers in PEC systems (Kesters et al., 2015) due to their wide availability in nature, high molar absorptivity and thermal stability (Lash, 2015). One such example is the use of porphyrin derivative, iron(III) meso-tetrakis (N-methylpyridinum-4-yl) porphyrin (FeTMPyP), in a PEC DNA biosensor (Zang et al., 2015). In this case, CdS quantum dots (QDs) modified with ssDNA formed the photo-active electrode. The porphyrin derivative specifically binds to dsDNA via groove interactions and reports the presence of dsDNA by catalyzing the oxidation of luminol to generate chemiluminescence. The photocurrent intensity of the biosensor did not show a detectable change after storage for 10 days, highlighting the stability of this biosensor. Porphyrin-based materials have been used in conjunction with inorganic semiconductors such as TiO_2_ (Shu et al., 2015), ZnO (Tu et al., 2011), and CdTe (Shi et al., 2016) to enhance the IPCE of these systems. A major difficulty in the wide spread use of porphyrin is that its chemical synthesis usually requires several steps with low overall yield, amounting to a high material cost (Kwon et al., 2003; MedKoo Biosciences, 2009).

Pdots are a class of emerging photoactive nanomaterials that offer incredible photostability (photobleaching quantum yield of 10^−7^-10^−10^), tailorable electrical and optical properties, minimal toxicity, good biocompatibility and ease of processing (Wang et al., 2016b; Li et al., 2017b; Shi et al., 2018b; Zhang et al., 2018c). Pdots and PPV derivatives have recently been used in PEC biosensors (Shi et al., 2018b; Zhou et al., 2018) due to their extraordinary light harvesting ability resulting from their large two-photon absorption cross sections (Feng et al., 2010). However, their use in biosensing architectures are required to be thoroughly explored because of their pH dependence and tunability of photoelectrochemical properties according to their molecular weights (Wu and Chiu, 2013; Yu et al., 2017a). Moreover, the photoelectrochemical properties of these materials are highly dependent on the electron transfer processes within the π-conjugated bonds (Wang et al., 2018a), requiring a fundamental understanding of these processes to be able to design highly efficient photoelectrochemical biosensing devices.

Organic semiconductors are attractive due to their tunability, low cost, metal free nature, and relative abundance; however, their low quantum efficiency often requires them to be coupled with other photoactive materials for creating photoelectrodes. Unlike the ionic or covalent bond in inorganic semiconductors, organic semiconductors are made of molecular units held together by weak van der Waals interactions (Dyer-Smith and Nelson, 2012). As a consequence, the mobility of the charge carriers in organic materials is generally smaller with longer response times upon excitation as compared to their inorganic counterparts (Dyer-Smith and Nelson, 2012) leading to smaller conductivity (Kus et al., 2018). Consequently, more research is needed toward creating *all organic* photoelectrodes that can be used in biosensing.

### Hybrid Semiconductors

Hybrid semiconductors are formed by: (i) coupling two inorganic semiconductors with different band gaps, (ii) complexation of organic and inorganic semiconductors (Zhao et al., 2014), and (iii) combining metal NPs (usually Au or Ag) with organic/inorganic semiconductors.

Coupling two or more inorganic semiconductors extends the absorption spectrum and increases the charge separation efficiency of the PEC system (Wen and Ju, 2016). As a result, hybrid material systems offer a higher photon-to-current conversion efficiency, which is important for enhancing the performance of biosensors (Zhao et al., 2014). For example, when TiO_2_ NPs are used with CdS QDs in insulin detection (Wen and Ju, 2016), CdTe is excited using visible light, and the photo-induced electrons are transferred from CdTe to the conduction band of TiO-_2_ NPs. Liu et al. (2017b) demonstrated improved sensitivity of microcystin detection by forming Z-scheme heterojunction of CdTe with Bi_2_S_3_ nanorods due to enhanced charge separation. Another strategy used to enhance the solar light harvesting efficiency of photoanodes composed of wide bandgap semiconductors (i.e. TiO_2_, ZnO, etc.) is upconversion (Chen et al., 2018). This is a type of anti-stoke process in which emission of higher energy photons is achieved by the absorption of two or more low-energy photons (Naik et al., 2017). Qiu et al. (2018) developed a hybrid upconverting structure where the narrow absorption band of TiO_2_ was improved by the use of core–shell NaYF_4_:Yb,Tm@TiO_2_ upconversion microrods. In this system, doped Yb^3+^ ions absorbed near-IR light, whereas the doped Tm^3+^ emitters produced the UV light through energy transfer upconversion (ETU). The upconverted photons were then absorbed by the TiO_2_ NPs, thereby, yielding effective IR-UV upconversion (Qiu et al., 2018). This core-shell NaYF_4_:Yb,Tm@TiO_2_ structure was used to detect carcinoembryonic antigen (CEA), which is a biomarker for colorectal cancer.

Complexation of organic and inorganic semiconductors are used to overcome the low charge conductivity, narrow absorption spectrum, and strongly bound excitons that are encountered in organic semiconductors (Malliaras, 2001; Coropceanu et al., 2007). This class of transducers typically demonstrates improved PEC response and physical and chemical properties compared to their purely organic or inorganic counterparts (Zhao et al., 2016; Hao et al., 2017a). This type of complexation was demonstrated using TiO_2_ mesocrystals (inorganic semiconductor) sensitized with polyethylenimine (organic polymer) (Dai et al., 2017). Polyethylenimine reduces the electron transport energy barrier of TiO_2_ mesocrystals by reducing the work function and thereby increasing the generated photocurrent. This type of performance enhancement was also seen in reduced graphene oxide (RGO)/CdS/ZnS photoelectrode, where a widened light absorption range, spatial separation of photogenerated electron-hole pairs, accelerated electron transfer, and reduction of surface defects resulting from the coupling of ZnS (wide bandgap, ~3.8 eV) and CdS (narrow bandgap, ~2.4 eV) was observed (Zhao et al., 2016). By using RGO further enhancement of the photocurrent was achieved as it facilitates the excited electron transfer from the conduction band (CB) of CdS to the CB of ZnO. Ultrafast electron transport was also realized by Matylitsky et al. (2009) and Wang et al. (2014c) by adsorbing an electron acceptor, methyl viologen (MV) on the surface of CdSe QDs. Here, MV acted as an electron relay and facilitated ultrafast electron transport in a timeframe of ~70 fs. The ability of MV in enhancing IPCE by working as an electron relay was exploited by Long et al. (2011) to demonstrate ultrafast electron transport in cysteine bioanalysis by using a MV coated CdS QD based system. Surface sensitization of a wide band gap semiconductor with an organic material such as a dye is an alternative method of creating efficient hybrid materials. Here, an increase in efficiency of the excitation due to the injection of electrons directly into the CB of the semiconductor from the excited dye and expansion of excitation wavelength range results in higher photocurrent generation (Lim et al., 2017; Ma et al., 2018; Yan et al., 2018). Yotsumoto Neto et al. (2016) demonstrated the usefulness of such hybrid materials in sensing L-Dopamine by using iron phthalocyanine (FePc) dye sensitized TiO_2_ system to enhance the PEC performance due to the charge transfer property of FePc. Additionally, the antioxidant character of FePc is also hypothesized to enhance stability of the biorecognition units used in this study by inhibiting PEC-induced damage to the attached biomolecules typically seen in the case of wide band gap semiconductors such as TiO_2_.

The coupling of inorganic/organic semiconductors with metal nanoparticles is increasingly used in PEC devices due to the ability of metal NPs such as gold, platinum and silver to enhance the photoresponse of the system through surface-plasmon resonance (SPR). Han et al. (2017a) detected α-fetoprotein (AFP), a key clinical indicator used for diagnosing primary liver cancer, using Au-ZnO flower-rods. Here, Au NPs enhanced the anodic photocurrent of ZnO flower-rods by extending the absorption to the visible region and by enhancing charge separation. Besides the SPR effect, Au NPs have been shown to improve the charge transfer properties of the substrate. For example, Lv et al. (2017) deposited Au NPs on p-CuBi_2_O_4_ electrodes to reduce the charge transfer resistance and hence enhance the cathodic photocurrent of p-CuBi_2_O_4_. Another hybrid photoelectrode used in biosensing is created by Au NP-decorated hematite (α-Fe_2_O_3_) nanorods (Li et al., 2018b). Despite being widely used in other PEC applications (such as PEC water splitting, photovoltaic cells etc.), α-Fe_2_O_3_ has been rarely used in PEC biosensing due to poor electron mobility and lack of binding with capture biomolecules. Enhancement of electron mobility was achieved by decorating α-Fe_2_O_3_ with Au NPs. Moreover, Au NPs were also used to covalently attach capture biomolecules to the photoactive electrodes. Au NPs have also been used as anchors to deposit probe DNA and for improving the photocurrent of g-C_3_N_4_-based photoelectrodes (Wang et al., 2018b). In this work, Au NPs are used with g-C_3_N_4_ for detecting zeatin, one of the main cytokines found in plant tissues responsible for promoting plant growth.

Hybrid semiconductors are gaining popularity as transduction elements for PEC biosensors owing to their performance enhancement resulting from the coupling of desirable qualities of its constituent materials and the unique properties generated as a consequence of their complexation. To be able to achieve higher IPCE from these hybrid structures, it is important to carefully choose the materials and control their composition and morphology. Controlling interfacial defects is an important consideration for designing hybrid PEC systems. A summary of the photoactive materials used in PEC biosensing is presented in Table 1.

**Table 1:** Properties of various photoactive species used in PEC biosensing.

**Photo electrode material**	**Excitation**	**Stability (“N” cycles, Rsd (%), period)**	**Base photo-current (A)**	**Enhanced absorption**	**Enhanced charge separation**	**Ease of functionalization**	**References**
**1.1 Inorganic Semiconductors**							
TiO_2_ NWs	Simulated sunlight	–	1.15 × 10^−3^	No	No	Yes TiO_2_ NWs functionalized with HRP via APTES-gluteraldehyde coupling.	Wang et al., 2014b
CdTe QD	Xenon lamp; 420 nm cut-off filter	Fairly stable.*N* = 17, ~Rsd ~9.16%, 360 s	~2.17 × 10^−7^	No	Decreased. Trap sites resulting from Ag_2_Te formation create new electron–hole recombination centers.	Yes; 3-Mercaptopropionic (MPA) modified CdTe via one pot synthesis; resultant carboxyl terminated surface.	Lin et al., 2016
CdTe QD	590 nm	Very stable.*N* = 8, Rsd ~0.9%, 275 s	~3.80 × 10^−7^	No	No	Yes; MPA modified CdTe via one pot synthesis; resultant carboxyl terminated surface.	Li et al., 2018a
CdTe QD	590 nm	Very stable.*N* = 15, no decrease in photocurrent, 425 s	~2.10 × 10^−7^ (anodic) ~1.20 × 10^−7^ (cathodic)	No	No	Yes; MPA modified CdTe via one pot synthesis; resultant carboxyl terminated surface.	Hao et al., 2017b
**1.2 Organic Semiconductors**							
g-C_3_N_4_	Visible	Very stable.*N* = 9, no significant decrease in photocurrent, 350 s	~3.00 × 10^−6^	Yes	Yes; MB intercalators following duplex formation at g-C_3_N_4_ enhance separation efficiency.	No	Da et al., 2018
FeTMPyP	Chemiluminescence	Very stable.Rsd ~4.3%. Long term stability over 10 days.	~2.50 × 10^−7^	Yes	No	No	Zang et al., 2015
PFBT Pdots (Polymer dots)	450 nm	Stable.*N* = 20, 400 s	~3.00 × 10^−8^	No	Yes; photogenerated electrons transferred to the proton in solution at low pH value.	Yes; Carboxylated surface obtained via synthesis procedure allows for easy immobilization of pDNA via amine-carboxyl interaction.	Shi et al., 2018b
**1.3 Hybrid Semiconductors**							
**1.3.1 Inorganic -Inorganic**							
CdTe-Bi_2_S_3_	Visible	Fairly stable.*N* = 8, Rsd~7.3%, 350 s	~4.00 × 10^−7^	Yes	Yes; Z-scheme heterojunction formation between CdTe and Bi_2_S_3._	No	Liu et al., 2017b
CdS/ZnS	Visible	Good long-term stability; 95.6% of its original value after 5 months	~3.00 × 10^−5^	Yes	Yes; Formation of heterojunction allowed the transfer of photogenerated electrons to ZnS conduction band.	Yes; CdS was modified by carboxyl groups which was used to attach with amine terminated DNA.	Shi et al., 2018a
Core–shell NaYF_4_:Yb,Tm@TiO_2_	Infrared	Fairly stable.*N* = 10, Rsd ~7.9%, 250 s	~1.25 × 10^−7^	Yes	Yes; Enhanced separation due to formation of Z-scheme heterojunction	No	Qiu et al., 2018
**1.3.2 Organic-Inorganic**							
TiO_2_-polyethylenimine mesocrystal	Visible	Very stable.*N* = 10, Rsd ~2.04%, 250 sExcellent long-term stability; 94.8% of initial value after 12 days	~4.00 × 10^−6^	Yes	Yes; Improved charge separation via ligand (OAM/PEI) modification	Yes; Organic ligand (OAM/PEI) modification confers the complex with reactive amine terminations capable of further chemical reaction.	Dai et al., 2017
CdS-MV	Xenon lamp	Poor stability.	~1.00 × 10^−7^	No	Yes; MV coating of CdS facilitates fast charge separation and a slow charge recombination upon irradiation.	Yes; Thioglycolic acid (TGA) capped CdS QDs formed via precipitation-based synthesis; resultant carboxyl terminated surface.	Long et al., 2011
TiO_2_-EPM	380-480 nm	Very stable.*N* = 10, Rsd ~2.04%, 400 s	~3.00 × 10^−6^	Yes	No	Yes; Amine and hydroxyl terminations on TiO_2_ conferred via EPM (ligand) conjugation.	Ma et al., 2018
**1.3.3 Metal NP -Inorganic/Organic**							
AuNP-ZnO FRs	Simulated sunlight	Very stable.*N* = 15, no decrease in photocurrent, 300 s	~2.50 × 10^−5^	Yes	Yes; Au NPs in the Au-ZnO FRs heterostructure enhances charge separation.	No	Han et al., 2017a
AuNP on p-CuBi_2_O_4_	>420 nm	Good long-term stability; 99.8% of its original value after 3 weeks	~4.00 × 10^−7^	No	Yes; Au NPs, as a front contact of p-CuBi_2_O_4_ enhance the efficiency of charge separation	Yes; Au NPs, as a front contact of *p*-CuBi_2_O_4_ allow conjugation with thiol terminated biomolecules.	Lv et al., 2017
Au NP/Graphene QD/g-C_3_N_4_ nanosheet	Xenon lamp	Very stable.*N* = 15, Rsd ~1.5%, 20 s	~4.5 × 10^−7^	No	Yes; g-C_3_N_4_ and GQD reduce the probability of recombination of photogenerated electrons and holes.	Yes; Au NPs allow conjugation with thiol terminated biomolecules	Wang et al., 2018b

*RSD represents relative standard deviation, which signifies the reproducibility of the sensor*.

## Transduction Mechanism

**Figure 3:**
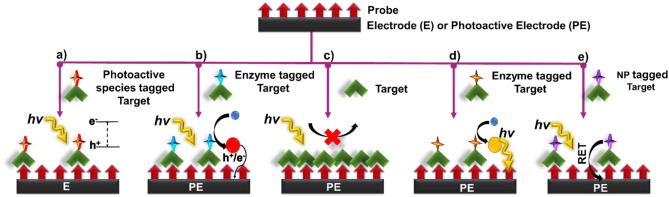
Principles of signal transduction in PEC biosensors **(a)** introduction of photoactive species, **(b)** generation of electron/hole donors, **(c)** use of steric hinderance, **(d)** in situ induction of light, and **(e)** resonance energy transfer.

Several signal transduction strategies have been proposed to translate a biorecognition event to a PEC signal. The signal of a PEC biosensor depends on the properties of the photoactive material, applied potential, light intensity, wavelength, and the type and concentration of the electron donor or acceptor (Zang et al., 2017). Depending on the mechanism chosen, the PEC biosensor operates in either *signal-on* or *signal-off* mode. In the former case, the PEC signal increases upon target recognition, and in the latter case it decreases (Zhao et al., 2018). In this review, we have categorized the signal generation strategies used in affinity-based biosensors as: (i) introduction of photoactive species, (ii) generation of electron/hole donors, (iii) use of steric-hindrance, (iv) *in situ* induction of light, and (v) resonance energy transfer (Figure 3). In this section, we discuss the recent biosensing reports categorized under these mechanisms.

### Introduction of Photoactive Species

In this signal transduction strategy, the photoactive material is incorporated into the target/probe complex in the form of a label, which enhances or quenches the PEC response. Signal modulation is initiated by bringing the label into the close proximity of a conductive/ photoactive substrate following a biorecognition event. Once in proximity to the appropriate substrate, electron hole pairs are generated at the surface of the photoactive material upon light excitation. These electron-hole pairs then take part in chemical reaction with the redox species in the electrolyte or interact with the underlying substrate to either generate a measurable signal or enhance an existing one (Figure 3a). Different types of photoactive species, such as semiconductor nanocrystals (Yue et al., 2013), metallic nanoparticles (Wang et al., 2019) and organic semiconductors such as g-C_3_N_4_, PFP (poly(9,9-bis(6′-(N,N,N,-trimethylammonium)hexyl) fluorene-co-alt-1,4-phenylene) bromide, etc. (Dai et al., 2016; Liu et al., 2018) have been commonly used as a signal label. In this strategy, it is crucial to (i) minimize the size of bioconjugated labels to reduce steric hindrance, (ii) decrease the effect of the label on the mass transport and complexation of the biomolecule, and (iii) diminish non-specific protein adsorption to develop labels that do not interfere with assay functionality. Semiconductor nanocrystals possess dimensions in the order of 1–100 nm, making them an excellent choice as PEC labels. The PEC signal of QDs in a complex environment stems from a myriad of factors such as the intensity of excitation source, the magnitude of applied bias potential, the absence/presence of electron donors and acceptors, as well as the inherent photophysical properties of QDs (Zhao et al., 2015a). These materials are particularly appealing owing to their tunable excitation spectrum resulting from quantum confinement, narrow and symmetrical emission spectrum, high quantum yield and good optical stability (Smith and Nie, 2010). Noble metal NPs are also commonly exploited for this approach (Golub et al., 2009; Hao et al., 2019). Plasmonic features of these particles such as intensive localized electric field generation in the near field, strong far-field light scattering, large absorption cross section in plasmonic resonance band, and light induced charge separation exhibited by these labels offer photocurrent modulation (Malekzad et al., 2017; Hao et al., 2019).

In this sensing scheme, in addition to the type of labels used, the electrode material greatly influences the assay design. Here, the electrode is typically constructed from wide bandgap semiconducting materials such as TiO_2_, g-C_3_N_4_, ZnO, p-CuBi_2_O_4_, and hematite (Fe_2_O_3_), where the photoactive label extends the absorption to the visible wavelength and improves the charge separation efficiency of the electrode. However, given that signal transduction is induced using a photoactive label, non-photoactive electrodes can also be used in these assays (Willner et al., 2001; Golub et al., 2009).

**Figure 4:**
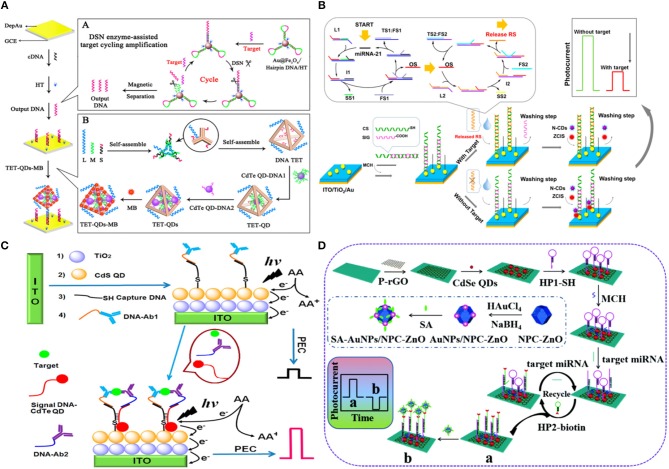
Introduction of QDs as Photoactive species: **(A)** Schematic Diagrams of PEC Biosensor for miRNA-141 detection using DSN enzyme-assisted target cycling amplification strategy and DNA TET-CdTe QDs-MB complex [Reprinted from Li et al. (2018a) with permission from American Chemical Society]. **(B)** Schematic illustration of the PEC detection of miRNA-21 by bringing photoactive N-doped carbon dots following hybridization of the target RNA [Reprinted from Chu et al. (2018) with permission from American Chemical Society]. **(C)** Schematic representation of ultrasensitive insulin detection based on CdTe QD labels brought into proximity of CdS/TiO_2_/ITO electrode upon affinity-based binding of CdTe QD labeled insulin target [Reprinted from Wen and Ju (2016) with permission from American Chemical Society]. **(D)** Schematic representation of the detection of miRNA-155 based on NPC-ZnO labeled target. Here, NPC-ZnO performs the role of electron scavenger, thus generating a signal-on response [Reprinted from Meng et al. (2019) with permission from American Chemical Society].

QDs are widely used as signal transduction reporters in PEC biosensors following the pioneering work of Willner et al. (2001) and Golub et al. (2009) in the early 2000s. In one of these works, ssDNA immobilized on a gold substrate was hybridized with CdS NP-tagged target DNA to a create a crosslinked CdS/DNA network (Yildiz et al., 2008; Golub et al., 2009). It was observed that the photocurrent emanating from these networks could be switched ‘on’ and ‘off’ using the light source through the photoejection of conduction-band electrons of CdS particles that were in contact with or at tunneling distances from the electrode using Ru(NH_3_)_6_ as an electron mediator. Using a similar strategy, Li et al. (2018a) reported an approach where they used DNA tetrahedron (TET) to deposit CdTe QDs and a methylene blue intercalator in the presence of the target analyte. Since the electrode was not photoactive, this system was operated at near-zero noise level and with a limit-of-detection of 17 aM and a linear range of 50 aM−50 pM in the presence of target miRNA-141 under light excitation (590 nm). When the DNA TET-CdTe QDs-MB complex was used as a signal probe, the PEC response (0.82 μA) was ~2.5-fold higher as compared to the PEC response based on the DNA TET-CdTe QDs complex alone (Figure 4A). In contrast to the previously discussed assays, the introduction of photoactive materials can also occur in the *absence* of the target analyte (Chu et al., 2018). In an assay of this type, the dsDNA capture probe contains a carboxyl-terminated ssDNA building block that is removed, through strand displacement, from the electrode upon target introduction. In the absence of the target strand, CuInS_2_/ZnS (ZCIS) QDs, and n-doped carbon dots are captured and increase the PEC signal under xenon lamp excitation (spectral range 200–1,200 nm) (Figure 4B). This biosensor exhibited a limit-of-detection of 0.31 pM with a linear range of 1 pM−100 nM in the presence of target miRNA-21. Furthermore, single base mismatch studies conducted using miRNA-21 (target), SM miRNA-21 and miRNA-141 showed ~4 × higher response in the case of target as compared to the interfering miRNAs, showcasing the excellent selectivity of this sensor. In addition to nucleic acid sensing, QDs are widely used in PEC biosensors created for protein analysis. In an assay of this type, the presence of insulin instigated the formation of an immunocomplex containing DNA-labeled antibody, insulin, secondary DNA labeled antibody and CdTe-labeled reporter DNA (Wen and Ju, 2016). CdTe induces a sensitization effect on the CdS/TiO_2_/ITO electrode, thereby enhancing the photocurrent under white light excitation (spectral range 200–1,200 nm) (Figure 4C). A limit-of-detection of 3 fM was exhibited by this sensor with a linear range of 10 fM−10 nM using insulin as the target. The fabricated sensor exhibited desirable long-term stability with no significant change in photocurrent following storage for 10 days and excellent selectivity when incubated with a solution containing interfering agents (IGF-1 and C-peptide). Micro-RNA (miRNA-155) detection has been shown by introducing Au NP functionalized N-doped porous carbon ZnO polyhedra (NPC-ZnO) on CdSe QD based photoelectrode (Meng et al., 2019). Following, miRNA hybridization with a hairpin structure probe, Au NP functionalized NPC-ZnO was brought close to the hybridized double-stranded RNA by using second hairpin DNA structure (Figure 4D). The NPC-ZnO is also photoactive and thereby generated a signal-on response under visible light excitation. This creative design strategy enabled ultrasensitive miRNA detection with a limit-of-detection of 49 aM (linear range of 0.1 fM−10 nM), which is much lower than the previously reported photoelectrochemical miRNA detection bioassays (Wen and Ju, 2016; Chu et al., 2018).

**Figure 5:**
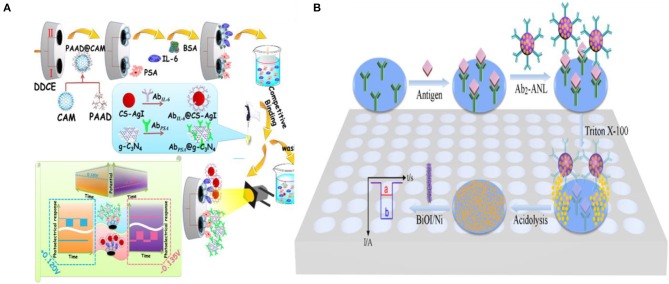
Introduction of photoactive species: **(A)** Schematic representation of two potentiometrically resolvable protein detection assays for PSA and human interleukin-6 involving the affinity-based binding of CS-AgI tagged IL-6 and CS-AgI tagged PSA [Reprinted from Dai et al. (2016) with permission from American Chemical Society]. **(B)** Liposomal PEC bioanalysis using photocathode and AgI/Ag; Reproduced with permission from [Reprinted from Yu et al. (2019) with permission from American Chemical Society].

Dai et al. (2016) demonstrated a multiplexed PEC immunoassay by using two different photoactive materials—graphitic carbon nitride (g-C_3_N_4_) which exhibited an anodic photocurrent and CS-AgI which exhibited a cathodic photocurrent—on a polyamidoamine dendrimer modified cube anatase TiO_2_ mesocrystal (PAAD@CAM) substrate (Figure 5A). A competitive immunoassay was designed to analyze PSA and IL-6 biomarkers using anti-PSA and anti-IL-6 antibodies labeled with g-C_3_N_4_ and CS-AgI, respectively. Application of different bias voltages allowed each of the complexes to be individually analyzed with IL-6 having a dynamic range of 10^−5^-90 pg mL^−1^ (3.3 × 10^−5^ pg mL^−1^ limit-of-detection) and PSA having a dynamic range of 10^−6^-90 ng mL^−1^ (3.3 × 10^−3^ pg mL^−1^ limit-of-detection).

Metal nanoparticles are used in combination with photoactive materials as signal reporters in PEC biosensors. In an assay of this kind, liposomes loaded with AgNP were labeled with IgG antibodies to detect IgG on a BiOI/Ni electrode (Yu et al., 2019). In a sandwich protein binding assay, the liposome-antibody conjugates were used to label the captured antigen. Upon binding, the Ag NPs were released using Triton X-100 and reacted with the p-type BiOI substrate to form an AgI/Ag/BiOI z-scheme heterojunction, enhancing the cathodic photocurrent of the electrode due to the reduction of dissolved O_2_ by AgI and transferring electrons from the conduction band of BiOI to the valence band of AgI through metallic Ag upon illumination (410 nm excitation light source) (Figure 5B). This assay demonstrated a limit-of-detection of 100 fg mL^−1^ and was linear in the 100 fg mL^−1^-100 ng mL^−1^ range.

As seen in the previous reports, target labeling provides the sensitivity and specificity that is needed for bioanalysis in complex biological samples. However, the introduction of photoactive species via labeling often impairs the rate and efficiency of bio-recognition, makes it difficult to perform quantitative analysis of biomolecular species in real time, and adds to the assay complexity due to the washing steps. An alternative method that can overcome some of the drawbacks of labeling is signal transduction via *in situ* generation of electron/hole donors, which is discussed in the following section.

### Generation of Electron/Hole Donors

**Figure 6:**
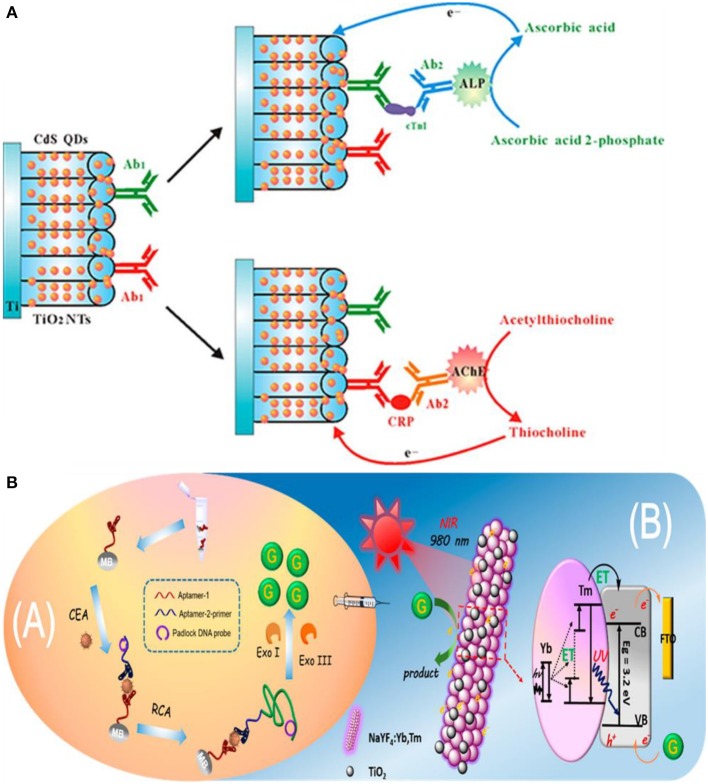
In situ generation of electron/hole donors: **(A)** Incorporation of dual enzyme tags for multiplexed cardiac troponin I (cTnI) and C-reactive protein (CRP) detection [Reprinted from Zhang et al. (2016a) with permission from American Chemical Society]. **(B)** Schematic Illustration of near infrared to ultraviolet light-mediated photoelectrochemical aptasensing for cancer biomarker detection and mechanism of signal generation in NaYF_4_:Yb,Tm@TiO_2_ photoactive electrode [Reprinted from Qiu et al. (2018) with permission from American Chemical Society].

In this approach, target introduction releases free electron/hole donors (scavenging species) that interact with the photoactive electrode surface, induce charge separation, and modulate the photocurrent. A common method to produce electron/hole donors is by using an enzyme (Zang et al., 2017) to generate hydrogen peroxide (H_2_O_2_) or ascorbic acid (AA). Alkaline phosphatase (ALP) is used in DNA and protein sandwich assays (Zhang et al., 2016b; Ju et al., 2019) to catalyze the conversion of ascorbic acid 2-phosphate (AAP) to ascorbic acid (AA) upon target binding. AA acts as a hole scavenger and increases the lifetime of photo-induced carriers, which enhances the PEC current (Ju et al., 2019). Using a similar approach, an assay incorporating dual enzyme tags for multiplexed PEC detection was developed to differentiate between two cardiac markers—cardiac troponin I (cTnI) and C-reactive protein (CRP) (Zhang et al., 2016b). ALP-tagged antibody was used for troponin T detection, and acetylcholine esterase (AChE)-tagged antibody was used for detecting C-reactive protein (Zhang et al., 2016b). These tags generate electron donating ascorbic acid (AA) and thiocholine (TC) by specifically catalyzing the hydrolysis of AAP or acetylthiocholine (ATC) (Figure 6A). Under visible light irradiation, the generated electron donors scavenge photoinduced holes at the surface of the CdS QDs/TiO_2_ electrode, inhibiting the recombination of the holes and electrons, thus enhancing the photocurrent. A linear range of 100 ng mL^−1^-0.1 mg mL^−1^ (a limit-of-detection of 50 ng mL^−1^) was exhibited for CRP, and a linear range of 1 ng mL^−1^-0.01 mg mL^−1^ (a limit-of-detection of 0.1 ng mL^−1^) was exhibited for cTnI.

In the previous assays, the biorecognition event, the generation of electron/hole donors, and signal measurement were performed on the electrode surface. However, it is possible to perform biorecognition and generate electron/hole donating species in solution and use the resultant species to modulate the PEC signal at an electrode surface. An assay of this type detects carcinoembryonic antigen (CEA) using a sandwich assay on the surface of magnetic beads (Qiu et al., 2018). Upon aptamer–CEA–aptamer reaction, the primer DNA on the terminus of the secondary aptamer initiates rolling circle amplification (RCA) reaction, resulting in the generation of long guanine (G) rich oligonucleotide strands (Figure 6B). Subsequent introduction of exonuclease I and III releases guanine (G) bases following digestion of the RCA product. The free guanine bases function as electron donors and enhance the photocurrent of the NaYF4:Yb,Tm@TiO_2_ microrod electrodes under near-infrared light excitation. The limit-of-detection of the assay for CEA target was 3 pg mL^−1^ with a linear range of 10 pg mL^−1^-40 ng mL^−1^. Furthermore, high specificity is demonstrated by this assay when tested against a complex mixture containing interferents such as PSA, TB, and human IgG.

In addition to using photoactive species and electron/hole donors separately, it is possible to combine these signal transduction mechanisms. For example, the synergistic effect of electron donor generation and photoactive species introduction was used to detect alpha-fetoprotein (AFP), which is a biomarker for liver cancer (Xu et al., 2015). In this assay, AFP-CdS-GOD complex was formed by conjugating AFP with CdS QD and glucose oxidase (GOD). In this work, chitosan which helps to covalently bind anti-AFP antibody was first deposited on the photoelectrode composed of ZnO inverse opal structure. Upon biorecognition with AFP-CdS-GOD, the photocurrent was enhanced. The enhancement of the photocurrent is attributed both to the increased absorption spectrum due to CdS QD and generation of H_2_O_2_ by GOD as electron donor. This bioassay showed a limit-of-detection of 0.01 ng mL^−1^ (linear range is 0.1–500 ng mL^−1^). Moreover, this assay showed good specificity against CEA, PSA and H_2_O_2_.

Although assays using electron/hole generation overcome the limitations encountered in labeled assays, they have some drawbacks that must be considered for using them in analyzing real-life samples. Enzymes that are typically used to induce the formation of local electron/hole donors are known for their instability, relatively low shelf life, and expensive reagent cost. Additionally, to ensure effective detection using this scheme, utmost importance must be paid to minimize interfering scavenging species found in native samples that may consume the locally generated electron/hole donors required to transduce the biorecognition events.

### Steric-Hindrance Based Assay

Introduction of a biomolecule at the biosensor surface can sterically hinder the access of electrolyte to the photoactive transducer to modulate the measured PEC current. This transduction approach is one of the simplest mechanisms for developing a biosensor because it usually does not require labeling steps following the target capture. However, most of these sensors operate in a signal-off fashion (Pang et al., 2017; Zang et al., 2017; Fu et al., 2018).

**Figure 7:**
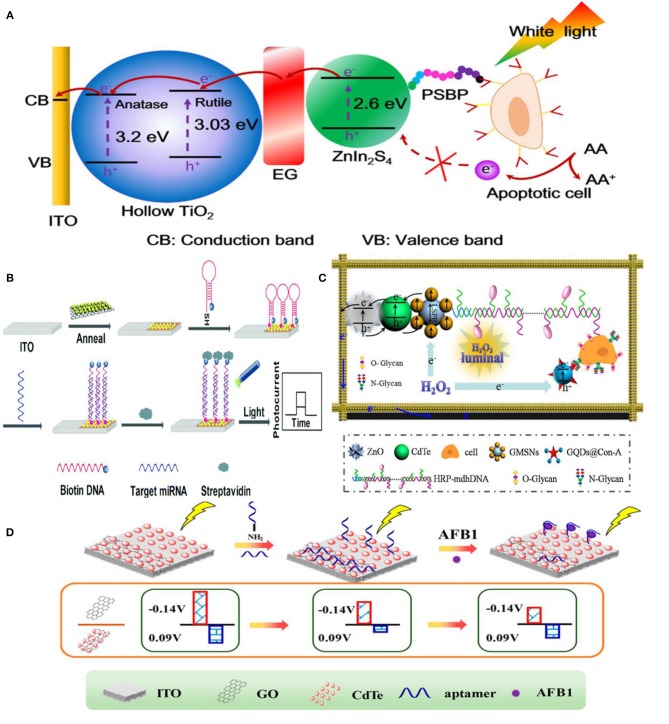
Steric-hindrance based biosensing: **(A)** Detection of apoptotic cells by TiO_2_/EG/ZnIn_2_S_4_ electrodes [Reprinted from Wu et al. (2018) with permission from American Chemical Society]. **(B)** Use of Au NPs in conjunction with a semiconductor (MoS_2_) to achieve higher photoresponse [Reprinted from Fu et al. (2018) with permission from The Royal Society of Chemistry]. **(C)** Detection of N glycan on ZnO_2_/CdTe/GMSNs electrode modified with GQD@conA [Reprinted from Ge et al. (2017) with permission from American Chemical Society]. **(D)** Two-channel approach for detecting AFB1 [Reprinted from Hao et al. (2017b) with permission from American Chemical Society].

In signal-off PEC biosensing, it is crucial to have a high photocurrent before target introduction because high concentrations of the target can completely diminish the PEC signal (Saha et al., 2018). Different approaches have been used to obtain high baseline PEC currents. Depositing photoactive materials into three-dimensional scaffolds such as wrinkled electrodes has been used to increase the photocurrent of PEC biosensors (Saha et al., 2018). The wrinkle electrodes showed 10 times higher photocurrent than a planar electrode composed of CdTe QDs. This wrinkled photoelectrode was used to detect single stranded DNA by simply hybridizing with the complementary sequence as a proof-of-concept. Moreover, it showed stable photocurrent following storage at 4°C in dark conditions for seven days and exhibited high specificity against single or multiple pair mismatch. Metal NPs are also deposited in combination with photoactive materials to enhance the photocurrent due to their plasmonic properties (Tu et al., 2016; Hao et al., 2018a). Fu et al. (2018) used Au NPs as a photoelectronic transfer promoter in photoactive molybdenum disulfide (MoS_2_) nanosheets to detect micro-RNA. To obtain further reduction of photocurrent, bulky biotin-streptavidin coupling was used in conjunction with probe hairpin DNA and target microRNA to increase the signal changes caused by steric hindrance. This PEC sensor presented a broad linear range of 10 fM−1 nM (limit-of-detection of 4.21 fM) (Figure 7B). Signal transduction using steric hindrance is ideally suited for cellular detection because the large size of cells compared to biomolecules enhances their steric hindrance effect. A signal-off sensor was constructed to rapidly detect early apoptotic cells using phosphatidylserine binding peptide (PSBP) bound to the surface of TiO_2_/Graphene/ZnIn_2_S_4_ photoelectrode as the biorecognition element (Wu et al., 2018). Here, the access of AA to the electrode surface was sterically hindered by the binding of the apoptotic cell decreasing the photocurrent. This biosensor exhibited an LOD of 3 cells mL^−1^ with a linear range of 1 × 10^3^-5 × 10^7^ cells mL^−1^ (Figure 7A). A paper-based cytosensor was reported for detecting breast cancer cells (MCF-7) constructed from ZnO spheres immobilized on Au nanorod-modified paper and sensitized with CdTe QDs and nanogold-assembled mesoporous silica nanoparticles (GMSNs) at their surface to create the photoactive portion of the biosensor (Ge et al., 2017). Multiple horseradish peroxidase (HRP) molecules and branched capture sites were then immobilized onto the GMSNs using double stranded DNA (Figure 7C). HRP was used in this assay to generate optical excitation through chemiluminescence. A signal decrease is observed upon capture of graphene quantum dot (GQD) labeled cancer cells as H_2_O_2_, the oxidant of luminol based chemiluminescence, is sterically hindered. These biosensors demonstrated a linear range of 63–1.0 × 10^7^ cells mL^−1^ and limit-of-detection of 21 cells mL^−1^. Lymphoblast (CCRF-CEM) cells were also detected based on steric hindrance in a PEC biosensor (Li et al., 2019). These cells were captured using hairpin DNA targeting overexpressed protein tyrosine kinase-7 on their surface. A decrease in PEC signal was exhibited on AgInS_2_ NPs photoelectrodes due to steric hindrance of AA. A limit-of-detection of 16 cells mL^−1^ and linear range of 1.5 × 10^2^-3.0 × 10^5^ cells mL^−1^ were demonstrated.

Another interesting signal transduction method involves the generation of a passivating compound as a result of target capture, which is used to decrease the PEC current generated on the photo-electrode (Zhuang et al., 2015; Qiu et al., 2018). An assay of this type combines biorecognition and isothermal target amplification in solution with signal modulation on the photoelectrode to detect T4 polynucleotide kinase (PNK), an important cellular regulator (Zhuang et al., 2015). In this detection scheme, a hairpin DNA (HP2) strand is phosphorylated upon the introduction of target PNK and is partially digested by λ-exo to yield an endogenous primer which initiates solution-based amplification generating DNA fragments. These DNA fragments activate the peroxidase-mimicking DNAzymes on the hairpin DNA probes immobilized on the photoelectrode (HP1) to catalyze the formation of insoluble precipitates at the electrode surface and attenuate the photocurrent response of the photoactive electrode. This target induced attenuation of current, and enabled PNK to be analyzed in the linear range of 2–100 mU mL^−1^. Zhang et al. (2018a) used this strategy to detect prostate specific antigen (PSA) on a CdS nanorod electrode. The presence of PSA led to the formation of a sandwich complex on Au NPs that contained DNAzyme concatamers that catalyzed the precipitation of 4-chloro-1-naphthol onto the photoactive electrodes in the presence of H_2_O_2_. The insoluble precipitate resulted in an attenuation of signal by inhibiting electron transfer from the electron donor AA to the photoelectrode. PSA detection was achieved in the 0.005 ng mL^−1^-50 ng mL^−1^ range with a limit-of-detection of 1.8 pg mL^−1^.

Multi-channel PEC biosensors operated based on steric hindrance have been developed for improved reliability (Zhang et al., 2017; Hao et al., 2018b; Hua et al., 2018). In an assay that uses sunlight instead of an external light source, a two-channel design enables the device to calibrate its photoresponse by considering the incoming sun light intensity (Hao et al., 2018b). In this assay, biorecognition event of aflatoxin B1 by the covalently bound aptamer with the underlying Ag/TiO_2_/3D nitrogen-doped graphene hydrogel (3DNGH) resulted in a decrease of photocurrent. The decrement of the photocurrent is attributed to the enhanced steric hindrance of the electrolyte (0.1 M PBS) to the electrode surface. Using this ratiometric approach an LOD of 2.5 × 10^−4^ ng mL^−1^ and linear range of 1.0 × 10^−3^-1.0 × 10^3^ ng mL^−1^ were achieved for the detection of aflatoxin B1 (AFB1), a highly toxic carcinogen mainly found in agricultural and sideline products such as cereals and dairy products. Building on this strategy, Hao et al. (2017b) developed another two channel device using CdTe-graphene oxide (GO) and CdTe photoelectrodes for detecting AFB1. A signal increase was observed on the CdTe-GO electrode because the aptamer was released from the electrode surface upon target capture, which improved the access of electrolyte (0.1 M PBS) to the photoelectrode (Figure 7D). A signal decrease was observed on the CdTe electrode upon target capture by the immobilized aptamer. Using this detection strategy, a limit-of-detection of 0.01 ng mL^−1^ and a linear range of 10 pg mL^−1^-100 ng mL^−1^ were observed. Compared to single channel PEC biosensors, this self-referencing design can provide better accuracy and reliability, thus providing a promising route for the future development of PEC biosensors.

Steric-hindrance based assays can also combined with other strategies, for example p53 (cell cycle regulator and tumor suppressor) detection has been shown by combining two detection strategies—(i) *in situ* generation of electron donors and (ii) the subsequent hindering of AA (Zhu et al., 2016). A protein G molecular membrane was used to immobilize ALP conjugate anti-p53 antibody on ordered TiO_2_ nanotubes containing Au NPs. ALP enzymatic reaction in the presence of AAP generates AA for scavenging the holes localized on Au NPs. In this system, immunocomplexation with the target (p53) decreases the photocurrent signal due to (i) increased steric hindrance caused by the immunocomplex and (ii) a change in dielectric permittivity of the Au NPs-TiO_2_ NTs interface following target capture, which in turn influences the energy coupling between Au NPs and TiO_2_ NTs. This sensor demonstrated a limit-of-detection of 0.05 ng mL^−1^ and a linear range of 20–100 ng mL^−1^ under 410 nm light illumination. Additionally, excellent selectivity was demonstrated by the immunoassay when faced with interfering agents such as glucose oxidase (GOD), prostate specific antigen (PSA), lysozyme (LZM), and thrombin. Furthermore, given that the average level of p53 in lung cancer patient serum samples is 0.55 ng mL^−1^, the limit-of-detection of this assay, along with its excellent selectivity point to its applicability for clinical use.

The simplicity of steric hinderance based signal transduction makes it appealing for use in PEC biosensing. However, due to its signal-off nature, this transduction method is associated with a higher incidence of false positives as compared to the other transduction methods discussed in this section. To overcome this, strategies such as multi-channel sensing with built-in calibration (Hao et al., 2017b) have been developed for more accurate and robust biosensing.

### *In situ* Induction of Light

**Figure 8:**
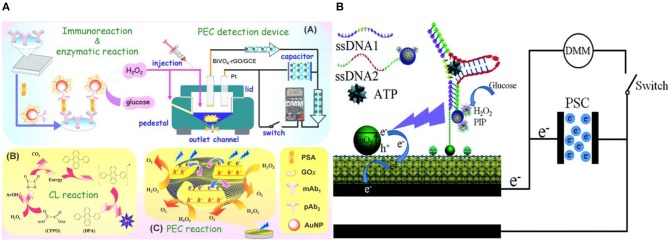
In situ generation of light. **(A)** PSA detection by coupling H_2_O_2_–triggered peroxyoxalate self-illuminated system with an external capacitor on the photoanode and digital multimeter as readout device [Reprinted from Shu et al. (2016) with permission from American Chemical Society]. **(B)** Schematic of the photocurrent generation mechanism in the modified paper sample zone of the Au-PWE under a CL light source [Reprinted from Wang et al. (2014a) with permission from Royal Society of Chemistry].

In conventional PEC biosensing, an external light-source is used for optical excitation, which imposes additional complexities for miniaturizing the biosensing platform (Tu et al., 2018). Elimination of the external light source is often achieved by employing chemiluminescence (CL) in the PEC biosensor for generating *in situ* light of various emission wavelengths (Ge et al., 2016b; Zang et al., 2017). In an assay of this type, prostate specific antigen (PSA) was captured and labeled in a sandwich assay with Au NPs modified with glucose oxidase (pAb_2_-AuNP-GOx) (Shu et al., 2016). GOx generates H_2_O_2_ enhancing the CL of the system, which in turn increases the photovoltage generated on the graphene oxide-doped BiVO_4_ photoelectrode (Figure 8A). This system showed a detection limit of 3 pg/mL and good specificity against CEA and AFP. A similar approach was used in a paper based PEC biosensor with porous Au/SnO_2_/rGO photoelectrodes for detecting ATP (Wang et al., 2014b). In this assay, the aptamer for ATP detection was split into two oligonucleotides. One of them (SSDNA1) were immobilized initially at the electrode surface and the other (SSDNA2) was conjugated with luminol and GOx into Fe_3_O_4_@Au NP. This nucleotide conjugate NP was brought to the electrode surface following the ATP introduction and thereby formed the complex shown in Figure 8B. Once GOx is bound to the electrode, it catalyzes the CL reaction by generating H_2_O_2_, which further reacts with the luminol. The sensing platform demonstrated a limit-of-detection of 0.025 pM with specificity against guanosine triphosphate (GTP), cytidine triphosphate (CTP), and uridine triphosphate (UTP). A proof-of-concept PEC DNA assay was also shown using this approach on CdS/MoS_2_ photoelectrodes (Zang et al., 2016). In this assay, target DNA is captured using an immobilized hairpin probe. Following the target-induced unfolding of the probe, the target DNA is displaced by a hemin-labeled DNA recycling probe. Hemin catalyzes luminol oxidation and generates CL, exciting the photoelectrode. This assay demonstrated a limit-of-detection of 0.39 fM and specificity against other forms of DNA (smDNA, tmDNA). The elimination of external light source makes this form of signal transduction appealing for the development of point-of-care devices. Yet another advantage of this method lies in the tunability of chemiluminescence by changing environmental factors such as the concentration of oxidizing species, environmental pH value, hydrophobicity of the solvent, and solution composition (Augusto et al., 2013; Fereja et al., 2013; He et al., 2014).

The signal transduction strategies elaborated thus far involved the reaction of photoactivated excitons with solution-based electron donors and acceptors to generate a measurable photocurrent, i.e., the signaling strategy established was based on the direct interfacial electron transfer between the photoactive material and ambient environment. The following section discusses signal transduction based on resonance energy transfer that involves the transfer of acquired *electronic energy* following photo-excitation.

### Resonance Energy Transfer

A powerful mechanism for modulating the PEC activity of the photoactive material in response to biorecognition is resonance energy transfer (RET) (Shu and Tang, 2017). In this approach, the biorecognition event serves as a mediator to bring a noble-metal NPs (Generally Au or Ag) to the proximity of the photoelectrode (Zang et al., 2017). Noble-metal NPs have very high extinction coefficients (Hartland, 2011) and can function as either signal quenchers (Zhao et al., 2011) or amplifiers (Han et al., 2015) depending on the distance between the metal and the other photoactive materials. If the absorption spectrum of the metal NP overlaps with the emission spectrum of the photoactive material, a significant portion of the exciton energy is transferred to the metal NPs, subsequently decreasing the photocurrent (Yun et al., 2005). However, when excited at plasmonic absorption wavelengths, a high electric field can surround the metal NPs and enhance the photocurrent generated by the photoactive material (Li et al., 2014a). In these assays, metal NPs are excited by the emission of the semiconductor already present in the electrode which is different from the approach where to enhance the PEC current, metal NPs are introduced during biomolecule recognition followed by external light as described in section Introduction of Photoactive Species.

**Figure 9:**
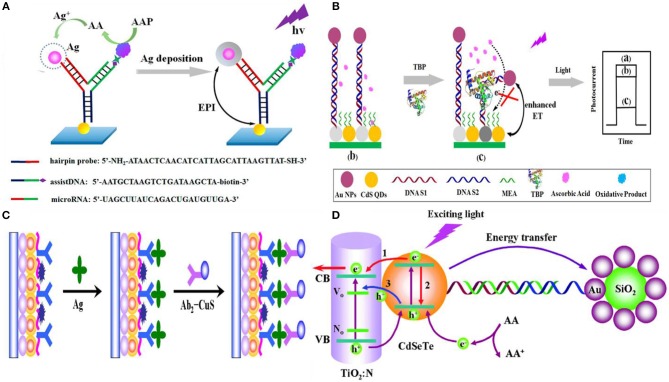
Resonance energy transfer based photoelectrochemical biosensors: **(A)** Energy transfer between CdS QDs and Ag NPs upon the ALP induced Ag deposition on Au NPs [Reprinted from Ma et al. (2016b) with permission from American Chemical Society]. **(B)** Tata binding protein bends the double-stranded DNA structure and brings CdS QD and Au NPs closer [Reprinted from Ma et al. (2016a) with permission from American Chemical Society]. **(C)** Schematic illustration of the signal-off sandwich type immunoassay was developed by using CuS nanocrystals as photocurrent quencher for early detection of CEA [Reprinted from Fan et al. (2016b) with permission from American Chemical Society]. **(D)** Thrombin detection using a PEC aptasensing platform based on exciton energy transfer between CdSeTe alloyed quantum dots and SiO_2_@Au nanocomposites. In this approach, RET significantly reduces the photocurrent, which is then quickly restored following the target's competitive binding and subsequent release of the metal NP tagged capture probe [Reprinted from Fan et al. (2015) with permission from The Royal Society of Chemistry].

Semiconductor QDs are commonly used as photoactive materials for electrodes in this approach because it is possible to tune their emission wavelength by varying their size. Mi-RNA detection has been shown by using RET between CdS QDs and Ag NPs under the illumination at a wavelength of 410 nm (Ma et al., 2016b). As shown in Figure 9A, target microRNA induces conformational change in the Au NP labeled hairpin probe deposited on CdS QD. ALP causes Ag deposition on the Au NPs, which significantly amplifies the signal decrease that is measured on the photoelectrode. This assay has demonstrated a detection limit of 0.2 fM with a linear range of 1 fM−100 pM. The same group has also shown DNA detection without the Ag deposition-induced amplification strategy (Zhang et al., 2016a) using CdS QDs and Ag NPs and achieved a limit-of-detection of 0.3 pM and a linear range of 1 pM−10 nM. Ma et al. (2016a) also used energy transfer between CdS QDs and Ag NPs to detect TATA-binding protein and achieved a limit-of-detection of 1.28 fM (linear range of 2.6 fM−512.8 pM). The CdS QDs used in this work have an emission peak around 530 nm which overlaps with the absorption peak of Au NPs. DNA hybridization was used to bring Au NP into the proximity of the semiconductor QDs (Figure 9B). Tata binding protein can further bend this dsDNA structure and bring Au NPs even closer to the CdS QDs. The TATA binding protein increases the signal attenuation due to the combined effect of RET and steric hindrance. This assay showed excellent selectivity against AFP, CEA, lysozyme, PSA, and thrombin.

Although Au and Ag NPs are the most popular materials used for RET-based PEC biosensing, other materials can also be used in these assays. For example, a signal-off sandwich-type immunoassay was developed by using CuS nanocrystals as the photocurrent quencher for early detection of CEA on CdSeTe@CdS:Mn-sensitized TiO_2_ NPs (Fan et al., 2016b). In this assay, CEA target antigens were captured using anti-CEA antibodies immobilized on the electrode surface, and a signaling antibody labeled with CuS was introduced to reduce the PEC current (Figure 9C). A limit-of-detection of 0.16 pg/mL with a linear range from 0.5 pg mL^−1^ to 100 ng mL^−1^ were achieved using this assay. The specificity of this assay was validated against PSA, AFP, carbohydrate antigen 19-9 and 15-3.

PEC biosensors that operate based on RET are highly sensitive. However, many of the RET biosensing assays reported to date are signal-off (Zang et al., 2017). It is possible to design a signal transduction method based on RET where the photocurrent is initially reduced and is *turned on* following target capture and the resultant removal of the metal NP (Shen et al., 2015; Liu et al., 2016). Thrombin detection was demonstrated using this approach where AuNP-decorated SiO_2_ nanoparticles were initially immobilized on CdSeTe QD-photoelectrodes through a dsDNA construct containing a thrombin-selective aptamer. Upon target capture, the signal diminishing Au NPs were removed from the electrode vicinity and a limit-of-detection of 2.8 fM with a linear range of 10 fM−50 pM was achieved (Fan et al., 2015) (Figure 9D). Consequently, it is possible to combine the high sensitivity of RET with reliability of signal-on sensing to create a high-performance biosensor. A summary of these recently published affinity-based PEC biosensor including their sensing mechanism, transduction approach and LOD are depicted in Table 2.

**Table 2:** Summary of the recent affinity-based PEC biosensor.

**Photoactive Material**	**Target**	**Sensing Approach**	**Transduction mechanism**	**LOD, linear range**	**References**
AgI/Ag/BiOI	IgG	Immunosensor, Signal on	Introduction of photoactive species	100 fg mL^−1^100 fg mL^−1^-100 ng mL^−1^	Yu et al., 2019
TiO_2_-CdS	Estradiol	Immunosensor, Signal off		2 pg mL^−1^5 pg mL^−1^-4 ng mL^−1^	Li et al., 2015
Donor–Acceptor-type PTB7-Th	Thrombin	Aptasensor, Signal on		34.6 fM100 fM−10 nM	Hu et al., 2018
CdS NPs	Oligonucleotides	DNA sensor		–	Willner et al., 2001
(PAAD)@ TiO_2_CAM, g-C_3_N_4_, CS-AgI	PSA, IL-6	Immunosensor, Signal polarity change		3.3 × 10^−5^ pg mL^−1^, 10–90 pg mL^−1^ (IL-6)3.3 × 10^−3^ pg mL^−1^, 10^−6^-90 ng mL^−1^ (PSA)	Dai et al., 2016
CdS QDs/NPC-ZnO	miRNA-155	DNAsensor, Signal on		49 aM0.1 fM−10 nM	Meng et al., 2019
CdS/TiO_2_, CdTe QDs	Insulin	Immunosensor, Signal on		3 fM10 fM−10 nM	Wen and Ju, 2016
Cationic polyfluorene derivative	Breast cancer cells (SKBR-3)	Cytosensor, Signal on		24 cells/mL^−1^1.0 × 10^2^-5.0 × 10^5^ cell mL^−1^	Liu et al., 2018
TiO_2_/ITO, Au NPs, [Ru(bpy)^3^]^2+^	DNA	Peptide sensor, Signal on		5.0 × 10^−3^ U mL^−1^10–50 U mL^−1^	Yan et al., 2016
TiO_2_/Au, CuInS_2_/ZnS (ZCIS) QDS	miRNA-21	Aptasensor, Signal on		0.31 pM1 pM−100 nM	Chu et al., 2018
CdTe, MB	miRNA-141	Aptasensor, signal on		17 aM50 aM−50 pM	Li et al., 2018a
MoS_2_/g-C_3_N_4_/black TiO_2_, Au NPs	miRNA	Aptasensor, Signal off		0.13 fM0.5–5,000 fM	Wang et al., 2019
GO/g-C_3_N_4_	Kanamycin	Aptasensor, Signal on		0.2 nM1–230 nM	Li et al., 2014b
TiO_2_	miRNA	DNAsensor, Signal Off-On		20 fM	Bettazzi et al., 2018
MB	miRNA	DNA sensor, Signal on		27 aM80 aM−10 pM	Hou et al., 2018
CdS/TiO_2_ NT	cTnI, CRP	Immunosensor, Signal on	Generation of electron/hole donor	0.1 ng mL^−1^, 1 ng mL^−1^-0.01 mg mL^−1^ (cTnI).50 ng mL^−1^, 100 ng mL^−1^-0.1 mg mL^−1^ (CRP)	Zhang et al., 2016b
NaYF_4_:Yb,Tm@TiO_2_	CEA	DNAsensor, Signal on		3.6 pg mL^−1^10 pg mL^−1^-40 ng mL^−1^	Qiu et al., 2018
Au NPs, MoS_2_	mi-RNA	DNAsensor, Signal off	Steric-hindrance	4.21 fM10 fM−1 nM	Zang et al., 2016
WS_2_, Au NP	MCF-7 cells	Aptasensor, Signal off		21 cells mL^−1^1 × 10^2^-5 × 10^6^ cells mL^−1^	Li et al., 2016
AuNP/g-C_3_N_4_	PNK	Aptasensor, Signal off		1 mU mL^−1^2 to 100 mU mL^−1^	Zhuang et al., 2015
Au NPs, TiO_2_	p53	Immunosensor, Signal off		0.05 ng mL20–100 ng mL^−1^	Zhu et al., 2016
Ag NPs, TiO_2_	AFB1	Aptasensor, Signal off		2.5 × 10^−4^ ng mL^−1^1.0 × 10^−3^-1.0 × 10^3^ ng mL^−1^	Hao et al., 2018b
Graphene loaded carbon QDs, g-C_3_N_4_	*E. coli*	Aptasensor, Signal off		0.66 cfu/mL2.9–2.9 × 10^6^ cfu/mL	Hua et al., 2018
CuO nanopyramid-island, AO	ALP	Immunosensor, Signal off		0.33 U L^−1^0.5–40.0 U L^−1^	Zhang et al., 2018b
TiO_2_ sensitized with ZnIn_2_S_4_	Early apoptotic HL-60 cells	Aptasensor, Signal off		3 cells mL^−1^1 × 10^3^-5 × 10^7^ cells mL^−1^	Wu et al., 2018
ZnO, Au nanorods, CdTe QD	MCF-7	Aptasensor, Signal off		21 cells mL^−1^63–1.0 × 10^7^ cells mL^−1^	Ge et al., 2017
TiO_2_/CdS:Mn, CuInS_2_ nanoflower	PSA	Aptasensor, Signal off		0.32 pg mL^−1^1 pg mL^−1^-100 ng mL^−1^	Fan et al., 2016a
CdS, Au NP	PSA	Aptasensor, Signal off		1.8 pg mL^−1^0.005–50 ng mL^−1^	Zhang et al., 2018a
AgInS_2_ NP	CCRF-CEM cells	Aptasensor, Signal off		16 cells mL^−1^1.5 × 10^2^-3.0 × 10^5^ cells mL^−1^	Li et al., 2019
CdS-MoS_2_ QD	DNA	DNA sensor, Signal on		0.39 fM1 fM−100 pM	Zang et al., 2016
TiO_2_-CdS:Mn	PSA	Immunosensor, Ratiometric		0.32 pg/mL1 pg/mL−100 ng/mL	Fan et al., 2016a
*p*-CuBi_2_O_4_-Au NP	AFP	Immunosensor, Signal off		14.7 pg/mL50 pg mL^−1^-20 ng mL^−1^	Lv et al., 2017
TiO_2_-EG-ZnIn_2_S_4_	Apoptotic cells HL-60	Immunosensor, Signal off		158 cells/mL1,000–50 × 10^7^	Wu et al., 2018
TiO_2_ Nanoneedls@MoO_3_	RAW264.7 macrophage cells	Immunosensor, Signal off		50 cells/mL50–1,500 cells/mL	Pang et al., 2017
ZnO spheres Au nanorod-CdTe QDs	Breast cancer cells (MCF-7)	Immunosensor, Signal off		21 cells/mL100–10^7^ cells per mL	Ge et al., 2017
t-mercaptopropionic acid capped AgInS_2_NP	Tumor cells	Aptamer sensor, Signal off		16 cells/mL1.5 × 10^2^-3.0 × 10^5^ cells/mL	Li et al., 2019
CdS-TiO_2_	CEA	Dnazyme sensor, Signal off	*In situ* induction of light	70 ag/mL70 ag/mL−500 fg/mL	Ge et al., 2016a
CdTe, CdTe-GO	AFB1	Aptasensor, Simultaneous signal on-off		10 pg mL^−1^10 pg mL^−1^-100 ng mL^−1^	Hao et al., 2017b
BiVO_4_-rGO-AuNP	PSA	Immunosensor, Signal on		3 pg/mL10 pg/mL−80 ng/mL	Shu et al., 2016
SnO_2_QD-RGO	ATP	Aptamer sensor		0.025 pM0.1 pM−100 nM	Wang et al., 2014a
CdS-Ag NP	Micro RNA/ss DNA	Aptamer sensor, Signal off	Resonance Energy Transfer	0.2 fM1 fM−100 pM0.3 pM1 pM−10 nM	Ma et al., 2016b; Zhang et al., 2016a
CdS-AuNP	Thrombin TATA binding protein	Aptamer sensor, Signal off		0.1 fM1 fM−10 pM50 fg/mL100 fg/mL−10 ng/mL	Ma et al., 2016a; Xu et al., 2016
TiO_2−x_-AuNP	ss DNA	DNA sensor, Signal on		0.6 pM1 pM−10 nM	Shu et al., 2018
CdSeTe-SiO_2_@Au	Thrombin	Aptamer sensor, Signal on		2.8 fM10 fM−50 pM	Fan et al., 2015

## Challenges and Future Perspectives

Affinity based biosensors using photoelectrochemistry as their transduction mechanism have garnered a lot of interest over the past decade due to their exceptional limit-of-detection. Biosensor development starts with considering the target analyte of interest, required limit-of-detection and specificity, interference caused by the native sample, and constraints of the operating environment (point-of-care, lab-based, resource poor compatible). This review aims at helping the reader choose the building blocks—materials and signal transduction mechanisms—of a PEC biosensor based on the constraints imposed by the application.

Inorganic and organic semiconductors are used as the photoactive building blocks for PEC biosensors. In PEC devices, photoactive materials are primarily chosen based on their efficiency in converting optical energy to electrochemical current or voltage. Using these materials in PEC *biosensing* adds additional requirements in terms of stability, size/structure, integration, cost, and functionalization. Given that photoactive materials used in PEC biosensing directly interact with nanoscale biomolecules, solution-processed photoactive *nanomaterials* that can be readily used as labels, reporters, or building blocks for the photoelectrode are primarily used in these systems. An important challenge with using these materials is their varying performance and stability in biosensing conditions that often require operation in complex biological environments and under stringent washing protocols. Affinity-based PEC biosensors operate by measuring signal changes that occur upon target binding; consequently, the lack of stability can cause non-target related signal changes, leading to false-positive or false-negative results. A key development toward the practical use of PEC biosensors involves incorporating *in situ* calibration measures in the PEC system to account for signal variations that are caused by the instability of photoactive materials. It is also critical to integrate functionalized photoactive materials into biosensing chips, strips, or cartridges using fabrication methods that are amenable to large volume processing.

We have reviewed the five most widely used signal transduction mechanisms used in PEC biosensing: introduction of photoactive species, generation of electron/hole donating species, use of steric hindrance, *in situ* induction of light, and resonance energy transfer. It is evident that it is possible to use any of these mechanisms to detect various classes of targets including nucleic acids, proteins, and cells. Additionally, a low limit-of-detection is possible using all of these assays. However, these assays vary greatly in terms of their fabrication and operation complexity. Ultimately, biosensing devices that are fabricated using scalable materials and methods have a higher chance for commercialization. Additionally, assays that can be operated using robust reagents in a simple and rapid one-pot manner have a higher chance for wide-scale adoption and success compared to those that require a sequence of washing and labeling steps. Consequently, choosing the right transduction method can be achieved by considering the collective requirements of a biosensing platform for use in real-life settings.

## Author Contributions

All authors listed have made a substantial, direct and intellectual contribution to the work, and approved it for publication.

### Conflict of Interest Statement

The authors declare that the research was conducted in the absence of any commercial or financial relationships that could be construed as a potential conflict of interest.

## References

[B1] Alizadeh N. Salimi A. (2018). Ultrasensitive bioaffinity electrochemical sensors: advances and new perspectives. Electroanalysis 30, 2803–2840. 10.1002/elan.201800598

[B2] Augusto F. A. de Souza G. A. de Souza Júnior S. P. Khalid M. Baader W. J. (2013). Efficiency of electron transfer initiated chemiluminescence. Photochem. Photobiol. 89, 1299–1317. 10.1111/php.12102 23711099

[B3] Bard A. J. Faulkner L. R. Leddy J. Zoski C. G. (1980). Electrochemical Methods: Fundamentals and Applications, Vol. 2. New York, NY: Wiley.

[B4] Bettazzi F. Laschi S. Voccia D. Gellini C. Pietraperzia G. Falciola L. (2018). Ascorbic acid-sensitized Au nanorods-functionalized nanostructured TiO_2_ transparent electrodes for photoelectrochemical genosensing. Electrochim. Acta 276, 389–398. 10.1016/J.ELECTACTA.2018.04.146

[B5] Chen E. Y. Milleville C. Zide J. M. O. Doty M. F. Zhang J. (2018). Upconversion of low-energy photons in semiconductor nanostructures for solar energy harvesting. MRS Energy Sustain. 5:E16 10.1557/mre.2018.15

[B6] Chen X. Mao S. S. (2007). Titanium dioxide nanomaterials: synthesis, properties, modifications, and applications. Chem. Rev. 107, 2891-2959. 10.1021/cr0500535 17590053

[B7] Chen Z. Deutsch T. G. Dinh H. N. Domen K. Emery K. Forman A. J. (2013). Efficiency definitions in the field of PEC, in Photoelectrochemical Water Splitting. SpringerBriefs in Energy (New York, NY: Springer), 7–16.

[B8] Cheng Y. Xiong P. Yun C. S. Strouse G. F. Zheng J. P. Yang R. S.. (2008). Mechanism and optimization of ph sensing using SnO_2_ nanobelt field effect transistors. Nano Lett. 8, 4179–4184. 10.1021/nl801696b 19367840 PMC2771949

[B9] Chu Y. Wu R. Fan G.-C. Deng A.-P. Zhu J.-J. (2018). Enzyme-free photoelectrochemical biosensor based on the co-sensitization effect coupled with dual cascade toehold-mediated strand displacement amplification for the sensitive detection of microRNA-21. ACS Sustain. Chem. Eng. 6, 11633–11641. 10.1021/acssuschemeng.8b01857

[B10] Coropceanu V. Cornil J. da Silva Filho D. A. Olivier Y. Silbey R. Brédas J. L. (2007). Charge transport in organic semiconductors. Chem. Rev. 107, 926–952. 10.1021/cr050140x 17378615

[B11] Da H. Liu H. Zheng Y. Yuan R. Chai Y. (2018). A highly sensitive VEGF165 photoelectrochemical biosensor fabricated by assembly of aptamer bridged DNA networks. Biosensors Bioelectronics 101, 213–218. 10.1016/j.bios.2017.10.032 29096358

[B12] Daghrir R. Drogui P. Robert D. (2013). Modified TiO_2_ for environmental photocatalytic applications: a review. Ind. Eng. Chem. Res. 52, 3581–3599. 10.1021/ie303468t

[B13] Dai H. Chen S. Li Y. Zeng B. Zhang S. Hong Z.. (2017). Photoelectrochemical biosensor constructed using TiO_2_ mesocrystals based multipurpose matrix for trypsin detection. Biosensors Bioelectronics 92, 687–694. 10.1016/J.BIOS.2016.10.028 27836612

[B14] Dai H. Zhang S. Hong Z. Lin Y. (2016). A potentiometric addressable photoelectrochemical biosensor for sensitive detection of two biomarkers. Anal. Chem. 88, 9532–9538. 10.1021/acs.analchem.6b02101 27584697

[B15] DeMiguel-Ramos M. Díaz-Durán B. Escolano J.-M. Barba M. Mirea T. Olivares J. (2017). Gravimetric biosensor based on a 1.3GHz AlN shear-mode solidly mounted resonator. Sensors Actuators B Chem. 239, 1282–1288. 10.1016/j.snb.2016.09.079

[B16] Deng W. Shen L. Wang X. Yang C. Yu J. Yan M.. (2016). Photoelectrochemical aptasensing. Trends Anal. Chem. 82, 307–315. 10.1016/j.trac.2016.06.02027088368

[B17] Devadoss A. Sudhagar P. Terashima C. Nakata K. Fujishima K. (2015). Photochemistry reviews photoelectrochemical biosensors: new insights into promising photoelectrodes and signal amplification strategies. J. Photochem. Photobiol. C Photochem. Rev. 24, 43–63. 10.1016/j.jphotochemrev.2015.06.002

[B18] Du J. Yang M. Zhang F. Cheng X. Wu H. Qin H. (2017). Enhanced charge separation of CuS and CdS quantum-dot-cosensitized porous TiO_2_-based photoanodes for photoelectrochemical water splitting. Ceramics Int. 44, 3099–3106. 10.1016/j.ceramint.2017.11.075

[B19] Dyer-Smith C. Nelson J. (2012). Chapter IE-2: organic solar cells, in Practical Handbook of Photovoltaics, 2nd Edn, eds McEvoy A. Markvart T. Castañer L. (Boston, MA: Academic Press), 543–569. 10.1016/B978-0-12-385934-1.00016-7

[B20] Fan D. Bao C. Khan M. S. Wang C. Zhang Y. Liu Q.. (2018). A novel label-free photoelectrochemical sensor based on N,S-GQDs and CdS co-sensitized hierarchical Zn_2_SnO_4_ cube for detection of cardiac troponin I. Biosensors Bioelectronics 106, 14–20. 10.1016/j.bios.2018.01.050 29414081

[B21] Fan G. C. Shi X. M. Zhang J. R. Zhu J. J. (2016a). Cathode photoelectrochemical immunosensing platform integrating photocathode with photoanode. Anal. Chem. 88, 10352–10356. 10.1021/acs.analchem.6b03473 27749029

[B22] Fan G. C. Zhu H. Du D. Zhang J. R. Zhu J. J. Lin Y. (2016b). Enhanced photoelectrochemical immunosensing platform based on CdSeTe@CdS:Mn core–shell quantum dots-sensitized TiO_2_ amplified by cus nanocrystals conjugated signal antibodies. Anal. Chem. 88, 3392–3399. 10.1021/acs.analchem.6b00144 26910366

[B23] Fan G. C. Zhu H. Shen Q. Han L. Zhao M. Zhang J. R.. (2015). Enhanced photoelectrochemical aptasensing platform based on exciton energy transfer between CdSeTe alloyed quantum dots and SiO_2_@Au nanocomposites. Chem. Commun. 51, 7023–7026. 10.1039/C5CC01935D 25804131

[B24] Feng X. Liu L. Wang S. Zhu D. (2010). Water-soluble fluorescent conjugated polymers and their interactions with biomacromolecules for sensitive biosensors. Chem. Soc. Rev. 39, 2411–2419. 10.1039/b909065g 20571669

[B25] Fereja T. H. Hymete A. Gunasekaran T. (2013). A recent review on chemiluminescence reaction, principle and application on pharmaceutical analysis. ISRN Spectroscopy 2013, 1–12. 10.1155/2013/230858

[B26] Fu B. Zhang Z. (2018). Periodical 2D photonic-plasmonic Au/TiOx nanocavity resonators for photoelectrochemical applications. Small 14:1703610. 10.1002/smll.201703610 29665208

[B27] Fu N. Hu Y. Shi S. Ren S. Liu W. Su S.. (2018). Au nanoparticles on two-dimensional MoS_2_ nanosheets as a photoanode for efficient photoelectrochemical miRNA detection. Analyst 143, 1705–1712. 10.1039/c8an00105g 29517787

[B28] Ge L. Wang W. Hou T. Li F. (2016a). A versatile immobilization-free photoelectrochemical biosensor for ultrasensitive detection of cancer biomarker based on enzyme-free cascaded quadratic amplification strategy. Biosensors Bioelectronics 77, 220–226. 10.1016/J.BIOS.2015.09.041 26409022

[B29] Ge S. Lan F. Liang L. Ren N. Li L. Liu H.. (2017). Ultrasensitive photoelectrochemical biosensing of cell surface N-glycan expression based on the enhancement of nanogold-assembled mesoporous silica amplified by graphene quantum dots and hybridization chain reaction. ACS Appl. Mater. Interfaces 9, 6670–6678. 10.1021/acsami.6b11966 28177218

[B30] Ge S. Liang L. Lan F. Zhang Y. Wang Y. Yan M. (2016b). Photoelectrochemical immunoassay based on chemiluminescence as internal excited light source. Sensors Actuators B Chem. 234, 324–331. 10.1016/j.snb.2016.04.166

[B31] Golub E. Pelossof G. Freeman R. Zhang H. Willner I. (2009). Electrochemical, photoelectrochemical, and surface plasmon resonance detection of cocaine using supramolecular aptamer complexes and metallic or semiconductor nanoparticles. Anal. Chem. 81, 9291–9298. 10.1021/ac901551q 19860374

[B32] Gong L. Zhao Z. Lv Y. F. Huan S. Y. Fu T. Zhang X. B.. (2015). DNAzyme-based biosensors and nanodevices. Chem. Commun. 51, 979–995. 10.1039/C4CC06855F 25336076

[B33] Gratzel M. (2001). Photoelectrochemical cells. Nature 414 338-344. 10.1038/35104607 11713540

[B34] Han D.-M. Jiang L.-Y. Tang W.-Y. Xu J.-J. Chen H.-Y. (2015). Photoelectrochemical determination of inorganic mercury ions based on energy transfer between CdS quantum dots and Au nanoparticles. Electrochem. Commun. 51, 72–75. 10.1016/j.elecom.2014.12.002

[B35] Han Z. Luo M. Chen L. Chen J. Li C. (2017a). A photoelectrochemical immunosensor for detection of α-fetoprotein based on Au-ZnO flower-rod heterostructures. Appl. Surface Sci. 402, 429–435. 10.1016/j.apsusc.2017.01.137

[B36] Han Z. Luo M. Chen L. Pan H. Chen J. Li C. (2017b). A photoelectrochemical biosensor for determination of DNA based on flower rod-like zinc oxide heterostructures. Microchim. Acta 184, 2541–2549. 10.1007/s00604-017-2257-5

[B37] Hao N. Hua R. Chen S. Zhang Y. Zhou Z. Qian J.. (2018a). Multiple signal-amplification via Ag and TiO_2_ decorated 3D nitrogen doped graphene hydrogel for fabricating sensitive label-free photoelectrochemical thrombin aptasensor. Biosensors Bioelectronics 101, 14–20. 10.1016/j.bios.2017.10.014 29031885

[B38] Hao N. Hua R. Zhang K. Lu J. Wang K. (2018b). A sunlight powered portable photoelectrochemical biosensor based on a potentiometric resolve ratiometric principle. Anal. Chem. 90, 13207–13211. 10.1021/acs.analchem.8b03218 30272953

[B39] Hao N. Lu J. Chi M. Xiong M. Zhang Y. Hua R. (2019). A universal photoelectrochemical biosensor for dual microRNA detection based on two CdTe nanocomposites. J. Mater. Chem. B 7, 1133–1141. 10.1039/C8TB03195A 32254781

[B40] Hao N. Zhang X. Zhou Z. Qian J. Liu Q. Chen S. (2017a). Three-dimensional nitrogen-doped graphene porous hydrogel fabricated biosensing platform with enhanced photoelectrochemical performance. Sensors Actuators B Chem. 250, 476–483. 10.1016/j.snb.2017.05.003

[B41] Hao N. Zhang Y. Zhong H. Zhou Z. Hua R. Qian J.. (2017b). Design of a dual channel self-reference photoelectrochemical biosensor. Anal. Chem. 89, 10133–10136. 10.1021/acs.analchem.7b03132 28929743

[B42] Hartland G. V. (2011). Optical studies of dynamics in noble metal nanostructures. Chem. Rev. 111, 3858–3887. 10.1021/cr1002547 21434614

[B43] He Y. He X. Liu X. Gao L. Cui H. (2014). Dynamically tunable chemiluminescence of luminol-functionalized silver nanoparticles and its application to protein sensing arrays. Anal. Chem. 86, 12166–12171. 10.1021/ac503123q 25421920

[B44] Hong Z. Jing L. Shusheng Z. (2015). Quantum dot-based photoelectric conversion for biosensing applications. Trends Anal. Chem. 67, 56–73. 10.1016/j.trac.2014.12.007

[B45] Hou T. Xu N. Wang W. Ge L. Li F. (2018). Truly immobilization-free diffusivity-mediated photoelectrochemical biosensing strategy for facile and highly sensitive microRNA assay. Anal. Chem. 90, 9591–9597. 10.1021/acs.analchem.8b02523 29991254

[B46] Hu T. Zheng Y. N. Li M. J. Liang W. B. Chai Y. Q. Yuan R. (2018). A highly sensitive photoelectrochemical assay with donor-acceptor-type material as photoactive material and polyaniline as signal enhancer. Anal. Chem. 90, 6096–6101. 10.1021/acs.analchem.8b00093 29676147

[B47] Hua R. Hao N. Lu J. Qian J. Liu Q. Li H.. (2018). A sensitive Potentiometric resolved ratiometric Photoelectrochemical aptasensor for *Escherichia coli* detection fabricated with non-metallic nanomaterials. Biosensors Bioelectronics 106, 57–63. 10.1016/j.bios.2018.01.053 29414089

[B48] Ikeda A. Nakasu M. Ogasawara S. Nakanishi H. Nakamura M. Kikuchi J. (2009). Photoelectrochemical sensor with porphyrin-deposited electrodes for determination of nucleotides in water. Organic Lett. 11, 1163–1166. 10.1021/ol900037q 19193047

[B49] Jiang D. Du X. Liu Q. Hao N. Wang K. (2019). MoS_2_/nitrogen doped graphene hydrogels p-n heterojunction: efficient charge transfer property for highly sensitive and selective photoelectrochemical analysis of chloramphenicol. Biosensors Bioelectronics 126, 463–469. 10.1016/j.bios.2018.11.018 30472443

[B50] Jiang Y. Tian B. (2018). Inorganic semiconductor biointerfaces. Nat. Rev. Mater. 3, 473–490. 10.1038/s41578-018-0062-3 PMC6815105 31656635

[B51] Ju Y. Hu X. Zang Y. Cao R. Xue H. (2019). Amplified photoelectrochemical DNA biosensor based on a CdS quantum dot/WS_2_ nanosheet heterojunction and hybridization chain reaction-mediated enzymatic hydrolysis. Anal. Methods 11, 2163–2169. 10.1039/C9AY00166B

[B52] Kadian S. Arya B. D. Kumar S. Sharma S. N. Chauhan R. P. Srivastava A. (2018). Synthesis and application of PHT-TiO_2_ nanohybrid for amperometric glucose detection in human saliva sample. Electroanalysis 30, 2793–2802. 10.1002/elan.201800207

[B53] Kang Z. Yan X. Wang Y. Bai Z. Liu Y. Zhang Z.. (2015). Electronic structure engineering of Cu_2_O film/ZnO nanorods array all-oxide p-n heterostructure for enhanced photoelectrochemical property and self-powered biosensing application. Sci. Rep. 5:7882. 10.1038/srep07882 25600940 PMC4298735

[B54] Kesters J. Verstappen P. Kelchtermans M. Lutsen L. Vanderzande D. Maes W. (2015). Porphyrin-based bulk heterojunction organic photovoltaics: the rise of the colors of life. Adv. Energy Mater. 5, 1–20. 10.1002/aenm.20150021826190957

[B55] Kirste R. Rohrbaugh N. Bryan I. Bryan Z. Collazo R. Ivanisevic A. (2015). Electronic biosensors based on III-nitride semiconductors. Ann. Rev. Anal. Chem. 8, 149–169. 10.1146/annurev-anchem-071114-040247 26048553

[B56] Kolesova E. P. Orlova A. O. Maslov V. G. Gun'ko Y. K. Cleary O. Baranov A. V. (2018). Photocatalytic properties of hybrid nanostructures based on nanoparticles of TiO_2_ and semiconductor quantum dots. Optics Spectroscopy 125, 99–103. 10.1134/S0030400X18070160

[B57] Komathi S. Muthuchamy N. Lee K. P. Gopalan A. I. (2016). Fabrication of a novel dual mode cholesterol biosensor using titanium dioxide nanowire bridged 3D graphene nanostacks. Biosensors Bioelectronics 84, 64–71. 10.1016/J.BIOS.2015.11.042 26611566

[B58] Kus M. Yilmaz Alic T. Kirbiyik C. Baslak C. Kara K. Akin Kara D. (2018). Chapter 24: synthesis of nanoparticles, in Handbook of Nanomaterials for Industrial Applications, ed C. Mustansar Hussain (Amsterdam: Elsevier), 392–429. 10.1016/B978-0-12-813351-4.00025-0

[B59] Kwon S. J. de Boer A. L. Petri R. Schmidt-Dannert C. (2003). High-level production of porphyrins in metabolically engineered *Escherichia coli*: systematic extension of a pathway assembled from overexpressed genes involved in heme biosynthesis. Appl. Environ. Microbiol. 69, 4875–83. 10.1128/aem.69.8.4875-4883.2003 12902282 PMC169110

[B60] Lash T. D. (2015). Benziporphyrins, a unique platform for exploring the aromatic characteristics of porphyrinoid systems. Organic Biomol. Chem. 13, 7846–7878. 10.1039/C5OB00892A 26061097

[B61] Li C. Du Y. Wang D. Yin S. Tu W. Chen Z. (2017a). Unique P-Co-N surface bonding states constructed on g-C_3_N_4_ nanosheets for drastically enhanced photocatalytic activity of H_2_ evolution. Adv. Funct. Mater. 27, 1–8. 10.1002/adfm.201604328

[B62] Li J. Lin X. Zhang Z. Tu W. Dai Z. (2019). Red light-driven photoelectrochemical biosensing for ultrasensitive and scatheless assay of tumor cells based on hypotoxic AgInS_2_ nanoparticles. Biosensors Bioelectronics 126, 332–338. 10.1016/j.bios.2018.09.096 30453133

[B63] Li J. Tu W. Li H. Han M. Lan Y. Dai Z.. (2014a). *In situ*-generated nano-gold plasmon-enhanced photoelectrochemical aptasensing based on carboxylated perylene-functionalized graphene. Anal. Chem. 86, 1306–1312. 10.1021/ac404121c 24377281

[B64] Li M. Xiong C. Zheng Y. Liang W. Yuan R. Chai Y. (2018a). Ultrasensitive photoelectrochemical biosensor based on DNA tetrahedron as nanocarrier for efficient immobilization of CdTe QDs-methylene blue as signal probe with near-zero background noise. Anal. Chem. 90, 8211–8216. 10.1021/acs.analchem.8b01641 29879840

[B65] Li R. Liu Y. Cheng L. Yang C. Zhang J. (2014b). Photoelectrochemical aptasensing of kanamycin using visible light-activated carbon nitride and graphene oxide nanocomposites. Anal. Chem. 86, 9372–9375. 10.1021/ac502616n 25219771

[B66] Li R. Liu Y. Yan T. Li Y. Cao W. Wei Q.. (2015). A competitive photoelectrochemical assay for estradiol based on *in situ* generated CdS-enhanced TiO_2_. Biosensors Bioelectronics 66, 596–602. 10.1016/j.bios.2014.12.002 25530540

[B67] Li R. Yan R. Bao J. Tu W. Dai Z. (2016). A localized surface plasmon resonance-enhanced photoelectrochemical biosensing strategy for highly sensitive and scatheless cell assay under red light excitation. Chem. Commun. 52, 11799–11802. 10.1039/C6CC05964C 27711382

[B68] Li Y. Zhang N. Zhao W. W. Jiang D. C. Xu J. J. Chen H. Y. (2017b). Polymer dots for photoelectrochemical bioanalysis. Anal. Chem. 89, 4945–4950. 10.1021/acs.analchem.7b00162 28384408

[B69] Li Z. Su C. Wu D. Zhang Z. (2018b). Gold nanoparticles decorated hematite photoelectrode for sensitive and selective photoelectrochemical aptasensing of lysozyme. Anal. Chem. 90, 961–967. 10.1021/acs.analchem.7b04015 29211440

[B70] Lim J. Bokare A. D. Choi W. (2017). Visible light sensitization of TiO_2_ nanoparticles by a dietary pigment, curcumin, for environmental photochemical transformations. RSC Adv. 7, 32488–32495. 10.1039/c7ra05276f

[B71] Lin Y. Zhou Q. Tang D. Niessner R. Yang H. Knopp D. (2016). Silver nanolabels-assisted ion-exchange reaction with CdTe quantum dots mediated exciton trapping for signal-on photoelectrochemical immunoassay of mycotoxins. Anal. Chem. 88, 7858–7866. 10.1021/acs.analchem.6b02124 27348353

[B72] Liu F. Zhang Y. Yu J. Wang S. Ge S. Song X. (2014). Application of ZnO/graphene and S6 aptamers for sensitive photoelectrochemical detection of SK-BR-3 breast cancer cells based on a disposable indium tin oxide device. Biosensors Bioelectronics 51, 413–420. 10.1016/J.BIOS.2013.07.066 24007750

[B73] Liu L. Hensel J. Fitzmorris R. C. Li Y. Zhang J. Z. (2010). Preparation and photoelectrochemical properties of CdSe/TiO^2^ hybrid mesoporous structures. J. Phys. Chem. Lett. 1, 155–160. 10.1021/jz900122u

[B74] Liu P. P. Liu X. Huo X. H. Tang Y. Xu J. Ju H. (2017a). TiO_2_-BiVO_4_ heterostructure to enhance photoelectrochemical efficiency for sensitive aptasensing. ACS Appl. Mater. Interfaces 9, 27185–27192. 10.1021/acsami.7b07047 28759199

[B75] Liu Q. Huan J. Dong X. Qian J. Hao N. You T. (2016). Resonance energy transfer from CdTe quantum dots to gold nanorods using MWCNTs/rGO nanoribbons as efficient signal amplifier for fabricating visible-light-driven “on-off-on” photoelectrochemical acetamiprid aptasensor. Sensors Actuators B Chem. 235, 647–654. 10.1016/j.snb.2016.05.154

[B76] Liu Q. Huan J. Hao N. Qian J. Mao H. Wang K. (2017b). Engineering of heterojunction-mediated biointerface for photoelectrochemical aptasensing: case of direct Z-scheme CdTe-Bi_2_S_3_ heterojunction with improved visible-light-driven photoelectrical conversion efficiency. ACS Appl. Mater. Interfaces 9, 18369–18376. 10.1021/acsami.7b04310 28497956

[B77] Liu S. He P. Hussain S. Lu H. Zhou X. Lv F.. (2018). Conjugated polymer-based photoelectrochemical cytosensor with turn-on enable signal for sensitive cell detection. ACS Appl. Mater. Interfaces 10, 6618–6623. 10.1021/acsami.7b18275 29368919

[B78] Liu X. Dai L. (2016). Carbon-based metal-free catalysts. Nat. Rev. Mater. 1:64 10.1038/natrevmats.2016.64

[B79] Liu Y. Yan K. Zhang J. (2015). Graphitic carbon nitride sensitized with CdS quantum dots for visible-light-driven photoelectrochemical aptasensing of tetracycline. ACS Appl. Mater. Interfaces 8, 28255–28264. 10.1021/acsami.5b08275 26574640

[B80] Long Y. T. Kong C. Li D. W. Li Y. Chowdhury S. Tian H. (2011). Ultrasensitive determination of cysteine based on the photocurrent of nafion-functionalized CdS-MV quantum dots on an ITO electrode. Small 7, 1624–1628. 10.1002/smll.201100427 21548084

[B81] Lv S. Zhang K. Lin Z. Tang D. (2017). Novel photoelectrochemical immunosensor for disease-related protein assisted by hemin/G-quadruplex-based DNAzyme on gold nanoparticles to enhance cathodic photocurrent on p-CuBi_2_O_4_ semiconductor. Biosensors Bioelectronics 96, 317–323. 10.1016/j.bios.2017.05.027 28525849

[B82] Ma H. Fan Q. Fan B. Zhang Y. Fan D. Wu D.. (2018). Formation of homogeneous epinephrine-melanin solutions to fabricate electrodes for enhanced photoelectrochemical biosensing. Langmuir 34, 7744–7750. 10.1021/acs.langmuir.8b00264 29884025

[B83] Ma Z. Y. Ruan Y. F. Xu F. Zhao W. W. Xu J. J. Chen H. Y. (2016a). Protein binding bends the gold nanoparticle capped DNA sequence: toward novel energy-transfer-based photoelectrochemical protein detection. Anal. Chem. 88, 3864–3871. 10.1021/acs.analchem.6b00012 26967949

[B84] Ma Z. Y. Xu F. Qin Y. Zhao W. W. Xu J. J. Chen H. Y. (2016b). Invoking direct exciton–plasmon interactions by catalytic Ag deposition on Au nanoparticles: photoelectrochemical bioanalysis with high efficiency. Anal. Chem. 88, 4183–4187. 10.1021/acs.analchem.6b00503 27023112

[B85] Maeda K. Wang X. Nishihara Y. Lu D. Antonietti M. Domen K. (2009). Photocatalytic activities of graphitic carbon nitride powder for water reduction and oxidation under visible light. J. Phys. Chem. C 113, 4940–4947. 10.1021/jp809119m

[B86] Malekzad H. Zangabad P. S. Mirshekari H. Karimi M. Hamblin M. R. (2017). Noble metal nanoparticles in biosensors: recent studies and applications. Nanotechnol. Rev. 6, 301–329. 10.1515/ntrev-2016-0014 29335674 PMC5766271

[B87] Malliaras G. G. (2001). Photovoltaic devices from organic semiconductors, in Encyclopedia of Materials: Science and Technology, eds Buschow K. H. J. Cahn R. W. Flemings M. C. Ilschner B. Kramer E. J. Mahajan S. Veyssière P. (Oxford: Elsevier), 6981–6986. 10.1016/B0-08-043152-6/01237-7

[B88] Matylitsky V. V. Dworak L. Breus V. V. Basché T. Wachtveitl J. (2009). Ultrafast charge separation in multiexcited CdSe quantum dots mediated by adsorbed electron acceptors. J. Am. Chem. Soc. 131, 2424–2425. 10.1021/ja808084y 19191491

[B89] MedKoo Biosciences (2009) Fe-TMPyP. Available online at: https://www.medkoo.com/products/15484

[B90] Meng L. Li Y. Yang R. Zhang X. Du C. Chen J. (2019). A sensitive photoelectrochemical assay of miRNA-155 based on a CdSe QDs//NPC-ZnO polyhedra photocurrent-direction switching system and target-triggered strand displacement amplification strategy. Chem. Commun. 55, 2182–2185. 10.1039/C8CC09411J 30699223

[B91] Mitchell K. Fahrenbruch A. L. Bube R. H. (1977). Photovoltaic determination of optical-absorption coefficient in CdTe. J. Appl. Phys. 48, 829–830. 10.1063/1.323636

[B92] Naik G. V. Welch A. J. Briggs J. A. Solomon M. L. Dionne J. A. (2017). Hot-carrier-mediated photon upconversion in metal-decorated quantum wells. Nano Lett. 17, 4583–4587. 10.1021/acs.nanolett.7b00900 28661675

[B93] Nelson J. (2002). Organic photovoltaic films. Curr. Opin. Solid State Mater. Sci. 6, 87–95. 10.1016/S1359-0286(02)00006-2

[B94] Ong W.-J. Tan L. L. Ng Y. H. Yong S. T. Chai S. P. (2016). Graphitic carbon nitride (g-C_3_N_4_)-based photocatalysts for artificial photosynthesis and environmental remediation: are we a step closer to achieving sustainability? Chem. Rev. 116, 7159–7329. 10.1021/acs.chemrev.6b00075 27199146

[B95] Özgür Ü. Alivov Y. I. Liu C. Teke A. Reshchikov M. A. Dogan S. (2005). A comprehensive review of ZnO materials and devices. J. Appl. Phys. 98:041301 10.1063/1.1992666

[B96] Pang X. Bian H. Su M. Ren Y. Qi J. Ma H.. (2017). Photoelectrochemical cytosensing of RAW264.7 macrophage cells based on a TiO_2_ nanoneedls@MoO_3_ array. Anal. Chem. 89, 7950–7957. 10.1021/acs.analchem.7b01038 28677958

[B97] Panneri S. Thomas M. Ganguly P. Nair B. N. Mohamed A. P. Warrier K. G. K. (2017). C3N4 anchored ZIF 8 composites: photo-regenerable, high capacity sorbents as adsorptive photocatalysts for the effective removal of tetracycline from water. Catal. Sci. Technol. 7, 2118–2128. 10.1039/c7cy00348j

[B98] Qiao Y. Li J. Li H. Fang H. Fan D. Wang W. (2016). A label-free photoelectrochemical aptasensor for bisphenol A based on surface plasmon resonance of gold nanoparticle-sensitized ZnO nanopencils. Biosensors Bioelectronics 86, 315–320. 10.1016/J.BIOS.2016.06.062 27387262

[B99] Qiu Z. Shu J. Tang D. (2018). Near-infrared-to-ultraviolet light-mediated photoelectrochemical aptasensing platform for cancer biomarker based on core-shell NaYF_4_:Yb,Tm@TiO_2_ upconversion microrods. Anal. Chem. 90, 1021–1028. 10.1021/acs.analchem.7b04479 29171254

[B100] Rim Y. S. Chen H. Zhu B. Bee S.-H. Zhu S. Li P. W. (2017). Interface engineering of metal oxide semiconductors for biosensing applications. Adv. Mater. Interfaces 4:1700020 10.1002/admi.201700020

[B101] Saha S. Chan Y. Soleymani L. (2018). Enhancing the photoelectrochemical response of DNA biosensors using wrinkled interfaces. ACS Appl. Mater. Interfaces 10, 31178–31185. 10.1021/acsami.8b12286 30192501

[B102] Sarkar S. Pal S. Sarkar P. (2012). Electronic structure and band gap engineering of CdTe semiconductor nanowires. J. Mater. Chem. 22:10716 10.1039/c2jm16810c

[B103] Shen Q. Han L. Fan G. Abdel-Halim E. S. Jiang L. Zhu J. J. (2015). Highly sensitive photoelectrochemical assay for DNA methyltransferase activity and inhibitor screening by exciton energy transfer coupled with enzyme cleavage biosensing strategy. Biosensors Bioelectronics 64, 449–455. 10.1016/j.bios.2014.09.044 25282398

[B104] Shi X. M. Fan G. C. Shen Q. Zhu J. J. (2016). Photoelectrochemical DNA biosensor based on dual-signal amplification strategy integrating inorganic-organic nanocomposites sensitization with λ-exonuclease-assisted target recycling. ACS Appl. Mater. Interfaces 8, 35091–35098. 10.1021/acsami.6b14466 27983802

[B105] Shi X. M. Fan G. C. Tang X. Shen Q. Zhu J. J. (2018a). Ultrasensitive photoelectrochemical biosensor for the detection of HTLV-I DNA: a cascade signal amplification strategy integrating λ-exonuclease aided target recycling with hybridization chain reaction and enzyme catalysis. Biosensors Bioelectronics 109, 190–196. 10.1016/j.bios.2018.03.023 29558733

[B106] Shi X. M. Mei L. P. Wang Q. Zhao W. W. Xu J. J. Chen H. Y. (2018b). Energy transfer between semiconducting polymer dots and gold nanoparticles in a photoelectrochemical system: a case application for cathodic bioanalysis. Anal. Chem. 90, 4277–4281. 10.1021/acs.analchem.8b00839 29528617

[B107] Shu J. Qiu Z. Lv S. Zhang K. Tang D. (2018). Plasmonic enhancement coupling with defect-engineered TiO_2−−x_: a mode for sensitive photoelectrochemical biosensing. Anal. Chem. 90, 2425–2429. 10.1021/acs.analchem.7b05296 29397702

[B108] Shu J. Qiu Z. Zhou Q. Lin Y. Lu M. Tang D. (2016). Enzymatic oxydate-triggered self-illuminated photoelectrochemical sensing platform for portable immunoassay using digital multimeter. Anal. Chem. 88, 2958–2966. 10.1021/acs.analchem.6b00262 26823201

[B109] Shu J. Qiu Z. Zhuang J. Xu M. Tang D. (2015). *In situ* generation of electron donor to assist signal amplification on porphyrin-sensitized titanium dioxide nanostructures for ultrasensitive photoelectrochemical immunoassay. ACS Appl. Mater. Interfaces 7, 23812–23818. 10.1021/acsami.5b08742 26451956

[B110] Shu J. Tang D. (2017). Current advances in quantum-dots-based photoelectrochemical immunoassays. Chemistry 12, 2780–2789. 10.1002/asia.201701229 28880459

[B111] Smith A. M. Nie S. (2010). Semiconductor nanocrystals: structure, properties, and band gap engineering. Accounts Chem. Res. 43, 190–200. 10.1021/ar9001069 19827808 PMC2858563

[B112] Soleymani L. Li F. (2017). Mechanistic challenges and advantages of biosensor miniaturization into the nanoscale. ACS Sensors 2, 458–467. 10.1021/acssensors.7b00069 28723192

[B113] Špačková B. Wrobel P. Bockova M. Homola J. (2016). Optical biosensors based on plasmonic nanostructures: a review. Proc. IEEE 104, 2380–2408. 10.1109/JPROC.2016.2624340

[B114] Su F. Mathew S. C. Lipner G. Fu X. Antonietti M. Blechert S. (2010). mpg-C_3_N_4_-Catalyzed selective oxidation of alcohols using O_2_ and visible light. J. Am. Chem. Soc. 132, 16299–16301. 10.1021/ja102866p 21043489

[B115] Tian J. Li Y. Dong J. Huang M. Lu J. (2018). Photoelectrochemical TiO_2_ nanotube arrays biosensor for asulam determination based on *in-situ* generation of quantum dots. Biosensors Bioelectronics 110, 1–7. 10.1016/j.bios.2018.03.038 29573621

[B116] Tomkiewicz M. Woodall J. M. (1977). Photoelectrolysis of water with semiconductor materials. J. Electrochem. Soc. 124, 1436–1440. 10.1149/1.2133669

[B117] Tong Z. Yang D. Li Z. Nan Y. Ding F. Shen Y.. (2017). Thylakoid-inspired multishell g-C_3_N_4_ nanocapsules with enhanced visible-light harvesting and electron transfer properties for high-efficiency photocatalysis. ACS Nano 11, 1103–1112. 10.1021/acsnano.6b08251 28032986

[B118] Tu W. Cao H. Zhang L. Bao J. Liu X. Dai Z. (2016). Dual signal amplification using gold nanoparticles-enhanced zinc selenide nanoflakes and P19 protein for ultrasensitive photoelectrochemical biosensing of microRNA in cell. Anal. Chem. 88, 10459–10465. 10.1021/acs.analchem.6b02381 27723295

[B119] Tu W. Lei J. Wang P. Ju H. (2011). Photoelectrochemistry of free-base-porphyrin-functionalized zinc oxide nanoparticles and their applications in biosensing. Chemistry 17, 9440–9447. 10.1002/chem.201100577 21678510

[B120] Tu W. Wang Z. Dai Z. (2018). Selective photoelectrochemical architectures for biosensing: design, mechanism and responsibility. Trends Anal. Chem. 105, 470–483. 10.1016/j.trac.2018.06.007

[B121] Wang A. Wang C. Fu L. Wong-Ng W. Lan Y. (2017). Recent advances of graphitic carbon nitride-based structures and applications in catalyst, sensing, imaging, and LEDs. Nano-Micro Lett. 9:2. 10.1007/s40820-017-0148-2 30393742 PMC6199047

[B122] Wang G. Yang X. Qian F. Zhang J. Z. Li Y. (2010). Double-sided CdS and CdSe quantum dot co-sensitized ZnO nanowire arrays for photoelectrochemical hydrogen generation. Nano Lett. 10, 1088–1092. 10.1021/nl100250z 20148567

[B123] Wang H. Wang Y. Zhang Y. Wang Q. Ren X. Wu D.. (2016a). Photoelectrochemical immunosensor for detection of carcinoembryonic antigen based on 2D TiO_2_ nanosheets and carboxylated graphitic carbon nitride. Sci. Rep. 6:27385. 10.1038/srep27385 27263659 PMC4893710

[B124] Wang L. Fernández-Terán R. Zhang L. Fernandes D. L. Tian L. Chen H.. (2016b). Organic polymer dots as photocatalysts for visible light-driven hydrogen generation. Angew. Chem. Int. Ed. 55, 12306–12310. 10.1002/anie.201607018 27604393

[B125] Wang M. Yin H. Zhou Y. Sui C. Wang Y. Meng X.. (2019). Photoelectrochemical biosensor for microRNA detection based on a MoS_2_/g-C_3_N_4_/black TiO_2_ heterojunction with Histostar@AuNPs for signal amplification. Biosensors Bioelectronics 128, 137–143. 10.1016/j.bios.2018.12.048 30660928

[B126] Wang Q. Ruan Y. F. Zhao W. W. Lin P. Xu J. J. Chen H. Y. (2018a). Semiconducting organic–inorganic nanodots heterojunctions: platforms for general photoelectrochemical bioanalysis application. Anal. Chem. 90, 3759–3765. 10.1021/acs.analchem.7b03852 29504756

[B127] Wang Y. Tang J. Zhou T. Da P. Li J. Kong B.. (2014a). Reversible chemical tuning of charge carriers for enhanced photoelectrochemical conversion and probing of living cells. Small 10, 4967–4974. 10.1002/smll.201401059 25044916

[B128] Wang Y. Xu J. Ma C. Li S. Yu J. Ge S. (2014b). A chemiluminescence excited photoelectrochemistry aptamer-device equipped with a tin dioxide quantum dot/reduced graphene oxide nanocomposite modified porous Au-paper electrode. J. Mater. Chem. B 2, 3462–3468. 10.1039/C4TB00233D 32261466

[B129] Wang Y. Zhao Y. Xu L. Han Z. Yin H. Ai S. (2018b). Photoelectrochemical apta-biosensor for zeatin detection based on graphene quantum dots improved photoactivity of graphite-like carbon nitride and streptavidin induced signal inhibition. Sensors Actuators B Chem. 257, 237–244. 10.1016/j.snb.2017.10.157

[B130] Wang Y.-F. Wang H.-Y. Li Z.-S. Zhao J. Wang L. Chen Q.-D. (2014c). Electron extraction dynamics in CdSe and CdSe/CdS/ZnS quantum dots adsorbed with methyl viologen. J. Phys. Chem. C 118, 17240–17246. 10.1021/jp5024789

[B131] Wen G. Ju H. (2016). Enhanced photoelectrochemical proximity assay for highly selective protein detection in biological matrixes. Analytical Chemistry, 88, 8339–8345. 10.1021/acs.analchem.6b02740 27464227

[B132] Willner I. Patolsky F. Wasserman J. (2001). Photoelectrochemistry with controlled DNA-cross-linked CdS nanoparticle arrays. Angew. Chem. 113, 1913–1916. 10.1002/1521-3757(20010518)113:10<1913::AID-ANGE1913>3.0.CO;2-P 11385656

[B133] Wu C. Chiu D. T. (2013). Highly fluorescent semiconducting polymer dots for biology and medicine. Angew. Chem. Int. Ed. 52, 3086–3109. 10.1002/anie.201205133 23307291 PMC5616106

[B134] Wu G. Hu Y. Liu Y. Zhao J. Chen X. Whoehling V.. (2015). Graphitic carbon nitride nanosheet electrode-based high-performance ionic actuator. Nat. Commun. 6:8258. 10.1038/ncomms8258 26028354 PMC4458862

[B135] Wu R. Fan G. C. Jiang L. P. Zhu J. J. (2018). Peptide-based photoelectrochemical cytosensor using a hollow-TiO_2_/EG/ZnIn_2_S_4_ cosensitized structure for ultrasensitive detection of early apoptotic cells and drug evaluation. ACS Appl. Mater. Interfaces 10, 4429–4438. 10.1021/acsami.7b16054 29327917

[B136] Xu F. Zhu Y. C. Ma Z. Y. Zhao W. W. Xu J. J. Chen H. Y. (2016). An ultrasensitive energy-transfer based photoelectrochemical protein biosensor. Chem. Commun. 52, 3034–3037. 10.1039/C5CC09963C 26790604

[B137] Xu J. Wang S. Wang G. N. Zhu C. Luo S. Jin L.. (2017). Highly stretchable polymer semiconductor films through the nanoconfinement effect. Science 355, 59–64. 10.1126/science.aah4496 28059762

[B138] Xu R. Jiang Y. Xia L. Zhang T. Xu L. Zhang S.. (2015). A sensitive photoelectrochemical biosensor for AFP detection based on ZnO inverse opal electrodes with signal amplification of CdS-QDs. Biosensors Bioelectronics 74, 411–417. 10.1016/j.bios.2015.06.037 26164013

[B139] Yan X. Li J. Yang R. Li Y. Zhang X. Chen Y. (2018). A new photoelectrochemical aptasensor for prion assay based on cyclodextrin and Rhodamine B. Sensors Actuators B Chem. 255, 2187–2193. 10.1016/j.snb.2017.09.030

[B140] Yan Z. Wang Z. Miao Z. Liu Y. (2016). Dye-sensitized and localized surface plasmon resonance enhanced visible-light photoelectrochemical biosensors for highly sensitive analysis of protein kinase activity. Anal. Chem. 88, 922–929. 10.1021/acs.analchem.5b03661 26648204

[B141] Yildiz H. B. Freeman R. Gill R. Willner I. (2008). Electrochemical, photoelectrochemical, and piezoelectric analysis of tyrosinase activity by functionalized nanoparticles. Anal. Chem. 80, 2811–2816. 10.1021/ac702401v 18324837

[B142] Yotsumoto Neto S. de Cássia Silva Luz R. Damos F. S. (2016). Visible LED light photoelectrochemical sensor for detection of L-Dopa based on oxygen reduction on TiO_2_ sensitized with iron phthalocyanine. Electrochem. Commun. 62, 1–4. 10.1016/j.elecom.2015.10.018

[B143] Yu J. Rong Y. Kuo C. T. Zhou X. H. Chiu D. T. (2017a). Recent advances in the development of highly luminescent semiconducting polymer dots and nanoparticles for biological imaging and medicine. Anal. Chem. 89, 42–56. 10.1021/acs.analchem.6b04672 28105818 PMC5682631

[B144] Yu K. J. Yan Z. Han M. Rogers J. A. (2017b). Inorganic semiconducting materials for flexible and stretchable electronics. npj Flexible Electronics 1:4 10.1038/s41528-017-0003-z

[B145] Yu S. Y. Mei L. P. Xu Y. T. Xue T. Y. Fan G. C. Han D. M.. (2019). Liposome-mediated *in situ* formation of AgI/Ag/BiOI Z-scheme heterojunction on foamed nickel electrode: a proof-of-concept study for cathodic liposomal photoelectrochemical bioanalysis. Anal. Chemi. 91, 3800–3804. 10.1021/acs.analchem.9b00352 30821438

[B146] Yue Z. Lisdat F. Parak W. J. Hickey S. G. Tu L. Sabir N.. (2013). Quantum-dot-based photoelectrochemical sensors for chemical and biological detection. ACS Appl. Mater. Interfaces 5, 2800–2814. 10.1021/am3028662 23547912

[B147] Yun C. S. Javier A. Jennings T. Fisher M. Hira S. Peterson S.. (2005). Nanometal surface energy transfer in optical rulers, breaking the FRET barrier. J. Am. Chem. Soc. 127, 3115–3119. 10.1021/ja043940i 15740151

[B148] Zang Y. Lei J. Hao Q. Ju H. (2016). CdS/MoS_2_ heterojunction-based photoelectrochemical DNA biosensor via enhanced chemiluminescence excitation. Biosensors Bioelectronics 77, 557–564. 10.1016/j.bios.2015.10.010 26476013

[B149] Zang Y. Lei J. Ju H. (2017). Principles and applications of photoelectrochemical sensing strategies based on biofunctionalized nanostructures. Biosensors Bioelectronics 96, 8–16. 10.1016/j.bios.2017.04.030 28454070

[B150] Zang Y. Lei J. Ling P. Ju H. (2015). Catalytic hairpin assembly-programmed porphyrin-DNA complex as photoelectrochemical initiator for DNA biosensing. Anal. Chem. 87, 5430–5436. 10.1021/acs.analchem.5b00888 25902380

[B151] Zhang K. Lv S. Lin Z. Li M. Tang D. (2018a). Bio-bar-code-based photoelectrochemical immunoassay for sensitive detection of prostate-specific antigen using rolling circle amplification and enzymatic biocatalytic precipitation. Biosensors Bioelectronics 101, 159–166. 10.1016/j.bios.2017.10.031 29065341

[B152] Zhang L. Sun Y. Liang Y. Y. He J. P. Zhao W. W. Xu J. J.. (2016a). Ag nanoclusters could efficiently quench the photoresponse of CdS quantum dots for novel energy transfer-based photoelectrochemical bioanalysis. Biosensors Bioelectronics 85, 930–934. 10.1016/j.bios.2016.06.018 27315518

[B153] Zhang N. Ma Z. Y. Ruan Y. F. Zhao W. W. Xu J. J. Chen H. Y. (2016b). Simultaneous photoelectrochemical immunoassay of dual cardiac markers using specific enzyme tags: a proof of principle for multiplexed bioanalysis. Anal. Chem. 88, 1990–1994. 10.1021/acs.analchem.5b04579 26841098

[B154] Zhang N. Ruan Y. F. Zhang L. B. Zhao W. W. Xu J. J. Chen H. Y. (2018b). Nanochannels photoelectrochemical biosensor. Anal. Chem. 90, 2341–2347. 10.1021/acs.analchem.7b04862 29283556

[B155] Zhang N. Shi X. M. Guo H. Q. Zhao X. Z. Zhao W. W. Xu J. J.. (2018c). Gold nanoparticle couples with entropy-driven toehold-mediated DNA strand displacement reaction on magnetic beads: toward ultrasensitive energy-transfer-based photoelectrochemical detection of miRNA-141 in real blood sample. Anal. Chem. 90, 11892–11898. 10.1021/acs.analchem.8b01966 30229657

[B156] Zhang Y. Hao N. Zhou Z. Hua R. Qian J. Liu Q.. (2017). A potentiometric resolved ratiometric photoelectrochemical aptasensor. Chem. Commun. 53, 5810–5813. 10.1039/C7CC01582H 28387390

[B157] Zhang Y. Luo J. Flewitt A. J. Cai Z. Zhao X. (2018d). Film bulk acoustic resonators (FBARs) as biosensors: a review. Biosensors Bioelectronics 116, 1–15. 10.1016/j.bios.2018.05.028 29852471

[B158] Zhang Y. Schnepp Z. Cao J. Ouyang S. Li Y. Ye J.. (2013). Biopolymer-activated graphitic carbon nitride towards a sustainable photocathode material. Sci. Rep. 3:2163. 10.1038/srep02163 23831846 PMC3703604

[B159] Zhao K. Yan X. Gu Y. Kang Z. Bai Z. Cao S.. (2016). Self-powered photoelectrochemical biosensor based on CdS/RGO/ZnO nanowire array heterostructure. Small 12, 245–251. 10.1002/smll.201502042 26618499

[B160] Zhao W. W. Wang J. Xu J. J. Chen H. Y. (2011). Energy transfer between CdS quantum dots and Au nanoparticles in photoelectrochemical detection. Chem. Commun. 47:10990. 10.1039/c1cc13952e 21909528

[B161] Zhao W. W. Wang J. Zhu Y. C. Xu J. J. Chen H. Y. (2015a). Quantum dots: electrochemiluminescent and photoelectrochemical bioanalysis. Anal. Chem. 87, 9520–9531. 10.1021/acs.analchem.5b00497 26023706

[B162] Zhao W. W. Xu J. J. Chen H. Y. (2014). Photoelectrochemical DNA biosensors. Chem. Rev. 114, 7421–7441. 10.1021/cr500100j 24932760

[B163] Zhao W. W. Xu J. J. Chen H. Y. (2015b). Photoelectrochemical bioanalysis: the state of the art. Chem. Soc. Rev. 44, 729–741. 10.1039/C4CS00228H 25223761

[B164] Zhao W. W. Xu J. J. Chen H. Y. (2017a). Photoelectrochemical enzymatic biosensors. Biosensors Bioelectronics 92, 294–304. 10.1016/j.bios.2016.11.009 27836594

[B165] Zhao W. W. Xu J. J. Chen H. Y. (2018). Photoelectrochemical immunoassays. Anal. Chem. 90, 615–627. 10.1021/acs.analchem.7b04672 29135236

[B166] Zhao X. Chaudhry S. T. Mei J. (2017b). Heterocyclic building blocks for organic semiconductors. Adv. Heterocyclic. Chem. 121, 133–171. 10.1016/bs.aihch.2016.04.009

[B167] Zheng Y. Liu J. Liang J. Jaroniec M. Qiao S. Z. (2012). Graphitic carbon nitride materials: controllable synthesis and applications in fuel cells and photocatalysis. Energy Environ. Sci. 5:6717 10.1039/c2ee03479d

[B168] Zhou H. Liu J. Zhang S. (2015). Quantum dot-based photoelectric conversion for biosensing applications. Trends Anal. Chem. 67, 56–73. 10.1016/J.TRAC.2014.12.007

[B169] Zhou X. Zhang P. Lv F. Liu L. Wang S. (2018). Photoelectrochemical strategy for discrimination of microbial pathogens using conjugated polymers. Chemistry 13, 3469–3473. 10.1002/asia.201800783 30084154

[B170] Zhu J. Xiao P. Li H. Carabineiro S. A. C. (2014). Graphitic carbon nitride: synthesis, properties, and applications in catalysis. ACS Appl. Mater. Interfaces 6, 16449–16465. 10.1021/am502925j 25215903

[B171] Zhu Y. C. Zhang N. Ruan Y. F. Zhao W. W. Xu J. J. Chen H. Y. (2016). Alkaline phosphatase tagged antibodies on gold nanoparticles/TiO_2_ nanotubes electrode: a plasmonic strategy for label-free and amplified photoelectrochemical immunoassay. Anal. Chem. 88, 5626–5630. 10.1021/acs.analchem.6b01261 27150939

[B172] Zhuang J. Lai W. Xu M. Zhou Q. Tang D. (2015). Plasmonic AuNP/g-C_3_N_4_ nanohybrid-based photoelectrochemical sensing platform for ultrasensitive monitoring of polynucleotide kinase activity accompanying DNAzyme-catalyzed precipitation amplification. ACS Appl. Mater. Interfaces 7, 8330–8338. 10.1021/acsami.5b01923 25837792

